# *Korarchaeota* Diversity, Biogeography, and Abundance in Yellowstone and Great Basin Hot Springs and Ecological Niche Modeling Based on Machine Learning

**DOI:** 10.1371/journal.pone.0035964

**Published:** 2012-05-04

**Authors:** Robin L. Miller-Coleman, Jeremy A. Dodsworth, Christian A. Ross, Everett L. Shock, Amanda J. Williams, Hilairy E. Hartnett, Austin I. McDonald, Jeff R. Havig, Brian P. Hedlund

**Affiliations:** 1 School of Life Sciences, University of Nevada, Las Vegas, Nevada, United States of America; 2 School of Earth and Space Exploration, Arizona State University, Tempe, Arizona, United States of America; 3 Department of Chemistry and Biochemistry, Arizona State University, Tempe, Arizona, United States of America; Missouri University of Science and Technology, United States of America

## Abstract

Over 100 hot spring sediment samples were collected from 28 sites in 12 areas/regions, while recording as many coincident geochemical properties as feasible (>60 analytes). PCR was used to screen samples for *Korarchaeota* 16S rRNA genes. Over 500 *Korarchaeota* 16S rRNA genes were screened by RFLP analysis and 90 were sequenced, resulting in identification of novel *Korarchaeota* phylotypes and exclusive geographical variants. *Korarchaeota* diversity was low, as in other terrestrial geothermal systems, suggesting a marine origin for *Korarchaeota* with subsequent niche-invasion into terrestrial systems. *Korarchaeota* endemism is consistent with endemism of other terrestrial thermophiles and supports the existence of dispersal barriers. *Korarchaeota* were found predominantly in >55°C springs at pH 4.7–8.5 at concentrations up to 6.6×10^6^ 16S rRNA gene copies g^−1^ wet sediment. In Yellowstone National Park (YNP), *Korarchaeota* were most abundant in springs with a pH range of 5.7 to 7.0. High sulfate concentrations suggest these fluids are influenced by contributions from hydrothermal vapors that may be neutralized to some extent by mixing with water from deep geothermal sources or meteoric water. In the Great Basin (GB), *Korarchaeota* were most abundant at spring sources of pH<7.2 with high particulate C content and high alkalinity, which are likely to be buffered by the carbonic acid system. It is therefore likely that at least two different geological mechanisms in YNP and GB springs create the neutral to mildly acidic pH that is optimal for *Korarchaeota*. A classification support vector machine (C-SVM) trained on single analytes, two analyte combinations, or vectors from non-metric multidimensional scaling models was able to predict springs as *Korarchaeota*-optimal or sub-optimal habitats with accuracies up to 95%. To our knowledge, this is the most extensive analysis of the geochemical habitat of any high-level microbial taxon and the first application of a C-SVM to microbial ecology.

## Introduction

A fundamental goal of ecology is to understand the distribution of organisms within the range of possible habitats and the factors controlling their distribution [1]. However, realization of this goal has been difficult for microbiologists, due to the complexity of natural microbial populations, problems accessing difficult-to-cultivate organisms, and the physicochemical complexity of environments in which they reside. Together, these challenges mandate tightly coordinated collection, processing, and analysis of biological, chemical, and physical data. Despite these challenges, a number of studies have examined spatial and temporal relationships between microbial community structure, both functionally and phylogenetically, and the geochemical environment [2], [3], [4], [5], [6]. Some studies have uncovered global patterns in microbial biodiversity that were unexpected. For example, Lozupone and Knight [7] parsed through >20,000 16S rRNA gene sequences from >100 cultivation-independent studies and showed that salinity and substrate type (planktonic versus sediment/soil-associated) dominate over other factors in controlling phylogenetic structure. Other studies have been more focused. Mathur et al. [4] showed strong correlations between microbiota and both substrate mineralogy and temperature in outflow channels of four acidic spring systems in Yellowstone National Park (YNP). *Hydrogenobaculum* was dominant in sulfur-rich sediments whereas uncultivated *Firmicutes* predominated in iron-rich sediments. Another study in YNP showed that geological history, not any physicochemical factor, controls the distribution of closely related *Sulfurihydrogenibium* phylotypes in 18 spring samples [6]. Population structure was delineated by ancient caldera boundaries, presumably because vicariant events are driven by greater opportunity for intra- rather than inter-caldera dispersal.

In this study, we carried out a census of *Korarchaeota*, a yet-to-be-isolated candidate phylum of *Archaea*. *Korarchaeota* were initially discovered as part of a diverse community of microorganisms in sediments from Obsidian Pool in YNP [8]. Originally, two phylotypes were described, pJP27 and pJP78, which were divergent on the level of a family (92% identity). Subsequently, Elkins et al. [9] obtained a complete genome sequence from a phylotype nearly identical to pJP27 from long (∼15 µm), ultrathin (<0.2 µm) *Korarchaeota* cells that were chemically and physically purified from a mixed culture that was originally inoculated with sediment from Obsidian Pool. Analysis of the “*Candidatus* Korarchaeum cryptofilum” genome suggested a physiology based on peptide fermentation coupled with proton reduction to H_2_, which is consistent with the sensitivity of *Korarchaeota* to H_2_ [9], [10]. The genome also suggested a dependency on other microorganisms because canonical pathways for biosynthesis of purines and several cofactors were absent, and supported the phylogenetic independence of *Korarchaeota* from the *Crenarchaeota* and *Euryarchaeota*.

Several subsequent studies have contributed to our understanding of the ecological niche of *Korarchaeota*. Small numbers of *Korarchaeota* 16S rRNA gene sequences were recovered in cultivation-independent censuses of a variety of geothermal habitats, both terrestrial [11], [12], [13], [14], [15], [16], [17], [18], [19] and marine [20], [21], [22], [23], [24], [25], [26], [27]. A study by Auchtung et al. [11] focused on defining the distribution of *Korarchaeota*, which resulted in the identification of nine *Korarchaeota* phylotypes in 8 of 41 YNP samples and a single sequence from a submarine sulfide chimney surface at the East Pacific Rise. *Korarchaeota* were not detected in a variety of cooler temperature settings. A study by Reigstad et al. [28] analyzed *Korarchaeota* abundance, diversity, biogeography, and biotic and abiotic habitat in 19 samples from Iceland and Kamchatka. Subsequently, another study by Auchtung et al. [19], demonstrated that *Korarchaeota* inhabiting Mutnovsky Volcano and the Uzon Caldera, roughly 260 km distant on the Kamchatka Peninsula, are closely related, but genetically distinct. Together, these studies suggested that *Korarchaeota* are exclusively thermophilic, expanded the geographical and geochemical range of the phylum, provided strong evidence of *Korarchaeota* endemism, and revealed extremely low phylogenetic diversity among *Korarchaeota* in terrestrial habitats. However, collectively, these studies incompletely identify the niche of *Korarchaeota* within geothermal habitats since relatively few geochemical measurements were made at the time and place of sampling.

Here, we built on the work of Auchtung et al. [11], [19] and Reigstad et al. [28] to define the habitat of *Korarchaeota* in terrestrial hot springs. To enhance our understanding of the precise geochemical habitats that support *Korarchaeota*, we expanded our sampling to a large number of geothermal features in two geographical regions, YNP and the U.S. Great Basin (GB), and paired quantitative biological sampling with an extensive analysis of geochemistry. The resultant data set included 107 samples, over 5,000 measurements of individual geochemical analytes, and 90 new *Korarchaeota* 16S rRNA gene sequences. Subsequently, we applied a variety of statistical tests to determine which factors correlated with *Korarchaeota* habitability and used a classification support vector machine (C-SVM) to develop models to predict whether a terrestrial geothermal habitat could support *Korarchaeota* based on geochemical data alone.

The results described here provide a robust description of *Korarchaeota* habitat in terrestrial geothermal ecosystems, strengthen evidence of biogeographic structure, reveal new phylogenetic diversity, provide the first ecological niche models, and complement the genomic work by Elkins et al. [9] in bringing the nature of *Korarchaeota* to light in the absence of axenic cultures.

## Materials and Methods

### Sample permits

All necessary permits were obtained for the described field studies. Samples in Yellowstone were collected under permit #5434 granted to ELS by the U.S. National Park Service, Yellowstone Office. Samples in the Great Basin were collected with permission from private land owners (Great Boiling Spring and Surprise Valley), the Bureau of Land Management (Grass Valley Spring, BLM Battle Mountain Regional Office), and the National Forest Service (Little Hot Creek, Inyo National Forest, Mammoth Lakes Office). No formal permits were required by private owners or public land managers for sampling these locations because disturbance due to sampling was deemed to be minimal. The field studies did not involve endangered or protected species.

### Sampling approach and bulk water physicochemical analysis

Springs were chosen to encompass a broad range of temperatures and pH. Temperature, pH and conductivity were measured with hand-held meters that were calibrated in the field prior to sampling (LaMotte 5 Series, Chestertown, MD or YSI Model 30, Yellow Springs, OH and WTW Model pH330i, Weilheim, Germany). Measurements were taken immediately before sediment sampling as close as possible to the precise sampling location.

Hydrothermal fluid was collected as close to the sampling site as possible prior to sediment sampling to avoid disrupting the sediment and altering the bulk water chemistry. Alkalinity, total ammonia, nitrate, nitrite, silica, total sulfide and dissolved oxygen were measured in the field colorimetrically (for GB springs LaMotte SMART 2 colorimeter, for YNP springs Hach DR/2400, Loveland, CO) (Table 1, 2, S1, S2). Some of these analyses are time sensitive due to gas dissolution and chemical/biological redox reactions, while others are more temperature sensitive. Water samples for measurement of alkalinity, total ammonia, nitrate, nitrite and silica were allowed to cool to ambient temperature for analysis. Alkalinity was determined by titration to pH 4.5. Ammonia was determined by using Nesslerization (LaMotte) or salicylate oxidation (Hach). Silica was determined by the measurement of molybdate-reactive silica with the heteropoly blue method in samples diluted with deionized water (DI). Nitrate plus nitrite was determined by cadmium reduction of nitrate and subsequent diazotization of nitrite. Nitrite was determined by diazotization without reduction of nitrate. Dissolved oxygen and sulfide were measured immediately after sampling. O_2_ measurements were made using the azide-modified Winkler method (LaMotte), the HRDO Accuvac ampule method (Hach, high range), or the Indigo Carmine method (Hach, low range); care was taken to minimize sample contact with the atmosphere. Sulfide was measured with the Pomeroy methylene blue method after dilution with ∼25°C DI (1∶3 or 1∶5) to prevent heat inactivation of reagents and to allow rapid analysis prior to oxidation.

**Table 1:** Description GB hot springs in which *Korarchaeota* 16S rRNA genes were detected.

Name^a^	Thermal Region	Thermal Area	GPS location (Datum: WGS84)	Sample type (mineralogy)^b^	Temp (°C)	pH
Great Boiling Spring 04c (GBS04c)	Great Boiling Sprgs	Great Boiling Sprgs	40°39.750′N, 119°21.985′W	gray sediment, green/brown mat	62.3	6.87
Sandy's Spring West source (SSW)	Great Boiling Sprgs	Mud Hot Springs	40°39.182′N, 119°22.496′W	gray/green sediment (S,I,K,Q,KF)	86.6	7.21
Sandy's Spring West (SSWcon1)	Great Boiling Sprgs	Mud Hot Springs	40°39.180′N, 119°22.500′W	gray sediment, green mat	70.0	7.85
Sandy's Spring West (SSWcon2)	Great Boiling Sprgs	Mud Hot Springs	40°39.172′N 119°22.485′W	green/brown flakey mat	58.6	8.28
Grass Valley Spring (GVS1)	Grass Valley	Grass Valley	39°56.462′N, 116°40.941′W	brown sediment	89.0	7.20
Hot Creek (HC-1)	Long Valley Caldera	Hot Creek	37°39.617′N, 118°49.745′W	light brown sediment	77.0	7.13
Little Hot Creek (LHC-1)	Long Valley Caldera	Little Hot Creek	37°41.436′N, 118°50.664′W	mixed sediment (S,I,K,Q,CA,PF,Z)	82.5	6.75
Little Hot Creek (LHC-3)	Long Valley Caldera	Little Hot Creek	37°41.456′N, 118°50.639′W	mixed sediment (I,C,S)	79.0	6.97
Little Hot Creek (LHC-4)	Long Valley Caldera	Little Hot Creek	37°41.436′N, 118°50.653′W	black sediment (S,I,K,CA)	78.7	6.85
Little Hot Creek (LHCcon1)	Long Valley Caldera	Little Hot Creek	37°41.432′N 118°50.642′W	black sediment	69.2	6.92
Little Hot Creek (LHCcon2)	Long Valley Caldera	Little Hot Creek	37°41.432′N, 118°50.632′W	sediment with black mat & filaments	59.9	7.20
Surprise Valley (SVX2)	Surprise Valley	Surprise Valley	41°32.075′N, 120°04.371′W	brown/gray aggregated sediment	83	8.49
Surprise Valley (SV2con3)	Surprise Valley	Surprise Valley	41°32.004′N, 120°04.327′W	gray sediment, green filaments	68.1	8.55

a Great Basin springs are typically named according to the “thermal region” or “thermal area” with an alphanumeric code. Short names in parenthesis are used in Table S1, S2.

b Minerals detected: S, smectite; I, illite; K, kaolinite; Q, quartz; KF, potassium feldspar; PF, plagioclase feldspar; CA, carbonate apatite; Z, zeolite clinoptilolite; C, calcite [12], [55].

**Table 2:** Description of YNP springs in which *Korarchaeota* 16S rRNA genes were detected.

Name^a^	Thermal Region	Thermal Area	GPS location (datum: WGS84)	Sample type (mineralogy)	Temp (°C)	pH
070714Y	Gibbon Geyser Basin	Sylvan Springs	44°41.927′N, 110°46.105′W	light brown sand	81.2	4.94
070714A	Gibbon Geyser Basin	Sylvan Springs	44°42.036′N, 110°45.931′W	grey sand	74.4	6.15
JRH060805G	Gibbon Geyser Basin	Sylvan Springs	44°42.031′N, 110°45.930′W	brown sediment with orange & black filaments	40.7	8.36
060809A	Washburn Springs	“Washburn Area”	44°45.879′N, 116°25.820′W	black sediment	85.9	5.88
070708X	Washburn Springs	“Washburn Area”	44°46.047′N, 110°25.807′W	black sand	76.1	5.70
070707T	Lower Geyser Basin	River Group	44°33.520′N, 110°50.643′W	black sediment	90.5	6.80
070707R	Lower Geyser Basin	River Group	44°33.530′N, 110°50.624′W	black sediment	74.3	8.50
070712EE	Lone Star Geyser	Channel Group	44°24.946′N, 110°48.578′W	beige sand	78.0	8.06
070712AA	Lone Star Geyser	Channel Group	44°24.903′N, 110°48.768′W	beige sand	73.3	6.50
070715V	Mud Volcano Area	“GOPA”	44°36.635′N, 110°26.314′W	mixed sediment	84.5	5.40
070715S	Mud Volcano Area	“GOPA”	44°36.603′N, 110°26.325′W	black sediment	71.3	6.35
070715T	Mud Volcano Area	“GOPA”	44°36.604′N, 110°26.325′W	black sediment	57.3	5.70
060804D	Mud Volcano Area	“GOPA”	44°36.640′N, 110°26.310′W	brown sediment	56.7	4.78
070712T	Calcite Springs	Calcite	not available	black sand	77.3	6.97

a Yellowstone springs are named according to the Yellowstone Research Coordination Network [53] whenever possible.

Water samples for ion chromatography (IC) and high-resolution inductively coupled plasma mass spectrometry (HR-ICP-MS) were collected in 60 mL high-density polypropylene bottles. Bottles for IC analysis were rinsed 3 times with NanoPure deionized water (DI) and soaked in DI for a minimum of 24 hours before being used. The IC samples were stored frozen until analysis. Concentrations of major cations (Na^+^, K^+^, Ca^2+^, Mg^2+^) and major anions (Cl^−^, NO_3_^−^, NO_2_^−^, Br^−^, SO_4_^2−^, PO_4_^3−^) were determined by ion chromatography. Anions were measured with a Dionex DX-600 ion chromatograph, consisting of a chromatography oven (LC 25), eluent generator (EG 40), electrochemical detector (ED 50), gradient pump (GP 50), and a Dionex AS-11-HC column. Cations were measured with a Dionex DX-120 ion chromatograph with a CS-12A column. Cations were separated isocratically using 18 mM methanesulfonic acid. Cation and anion samples were injected twice using a Dionex autosampler (AS40), and reported concentrations are averages of the two replicates. Analytical uncertainties are ±5% relative standard deviation (RSD) or better [29]. Sample dilutions were made with NanoPure^tm^ deionized water in a HEPA-filtered hood.

Bottles for HR-ICP-MS analysis were soaked in 10% nitric acid for a minimum of 24 hours, then rinsed 3 times with NanoPure DI and dried in a laminar flow hood with a HEPA air filter. The bottles were spiked with 200 µL of ultra-pure nitric acid (EMD, Omni Trace Ultra) to ensure sample preservation with no precipitation of metals. A suite of 52 minor and trace elements were quantified using a Thermo ELEMENT 2 single-collector double-focusing magnetic-sector inductively coupled plasma mass spectrometer. The instrument was operated at a cooling gas flow rate of 16 L min^−1^, auxiliary gas flow rate of 0.85 L min^−1^, and sample gas flow rate of 1.1 L min^−1^, with 3 by 3 runs/passes and sample uptake rate of 400 µL min^−1^. The resolution mode (low, medium, or high) used depends on the target metal. An internal standard of 1 ppb indium is used. Calibration standards were diluted from a 10 ppm stock solution to 0.5, 1, 5, 10 and 30 ppb. Nitric acid (EMD, Omni Trace Ultra) was used in the dilution. Certified reference materials from the National Institute of Standards and Technology (NIST) were measured before sample analysis, as well as after every 10 samples, to ensure measurement accuracy. Certified reference materials included NIST standard reference material (SRM) 1640 for inorganic trace elements in natural waters, NIST SRM 1643e trace elements in waters, and NRC CNRC SLRS4 river water for trace metals. Analytical uncertainties (%RSD) for low-resolution analyses are less than 3%, and <5% RSD for medium and high-resolution analyses (Table S1).

Sediment samples for DNA extraction and mineralogical analysis were collected at the sediment/water interface (top ∼1 cm). Collection utilized a spatula that was sterilized with 10% bleach. Sediments were scooped directly into sterile collection tubes (microcentrifuge tubes or polypropylene tubes) or into a sterile aluminum pie pan, where it was homogenized before distribution into collection tubes. Sediment from YNP springs was stored on ice in the field before storage (within 6 hrs.) at −20°C, whereas GB sediments were placed immediately on dry ice before long-term storage at −80°C.

### Particulate C and N geochemistry

Hot spring sediments were stored frozen until analysis. Weight percent total carbon (%TC), organic carbon (%OC) and total nitrogen (%TN), as well as the stable carbon isotopic composition (δ^13^C) were determined by high-temperature combustion on a Costech elemental analyzer coupled to a Thermo Finnigan DeltaPlus isotope ratio mass spectrometer (IRMS) using standard methods [30], [31], [32]. Briefly, samples were thawed, dried to a constant weight, homogenized in a ball mill and weighed (∼10–30 mg) into tared silver capsules. Sub-samples for total carbon (TC) and total N (TN) were analyzed directly; sub-samples for organic carbon (OC) were acidified with 6N HCl prior to analysis to remove inorganic carbon. Weight percent inorganic carbon was determined as the difference between total carbon and organic carbon (e.g., C_Inorganic_ = C_total_−C_Organic_).

### DNA extraction and evaluation of quality of DNA

Genomic DNA was extracted from sediment samples by using a bead-beating/SDS lysis approach using the QBIOgene FastDNA SPIN kit for soil (Irvine, CA). The manufacturer's general protocol was followed for all steps, with the specific extraction parameters described below. Cells in 0.5±0.1 g of sediment were lysed by milling the sample using the FastPrep instrument 4 times at setting 4.5 for 30 s with 5 min incubation on ice between each cycle.

PCR using “universal” primers for bacterial and archaeal 16S rRNA gene fragments was used to evaluate the suitability of the DNA for PCR. Following that screen, only DNA extracts yielding products using universal primers were deemed suitable for *Korarchaeota*-specific PCR. The initial screen employed primers 8aF [33] and 1406uR [34] for *Archaea* or 9bF [33] and 1406uR for *Bacteria*. Cycling conditions were as follows: initial denaturation at 96°C for 5 min; 35 cycles at 95°C for 30 s, 55°C for 30 s, and 72°C for 1.5 min; and final extension at 72°C for 5 min. Each 25 µl reaction included 1× GoTaq® Green Reaction Buffer (pH 8.5, 1.5 mM MgCl_2_; Promega, Madison, WI), dNTP mix (80 µM each; Promega), primers (200 nM each), GoTaq® DNA Polymerase (0.65 U; Promega), and 1 µl template DNA solution. *Escherichia coli* cell lysate was used for the positive control for each bacterial PCR and *Halostagnicola* sp. SL1.60 [35] was used as a positive control for each archaeal PCR. Reactions without template DNA were set up with each PCR to serve as negative controls. DNA was used directly from extracts in most cases; however, a 10-fold dilution with sterile water was necessary in a few instances because the extracts contained PCR inhibitors whose activities could be overcome by dilution. 99 of 107 DNA extracts yielding positive PCR results with “universal” primer sets for *Archaea* and/or *Bacteria* were used as templates for *Korarchaeota*-specific 16S rRNA gene PCR as described below.

### Korarchaeota-specific PCR

Primers 236F [10] and Kor1236R [11] were chosen for *Korarchaeota*-specific PCR for the study. The protocol for *Korarchaeota*-specific 16S rRNA gene PCR was modified from the 16S rRNA gene PCR protocol by optimizing the annealing temperature using a Mastercycler Gradient Thermal Cycler (Eppendorf) and experimenting with primer concentration, cycle number, and additions of bovine serum albumin (BSA). Optimal conditions were identical to the standard 16S rRNA gene PCR protocol, except that the number of cycles was increased to 40, the primer concentration was doubled to 400 nM each, and the annealing temperature was increased to 69°C. Comparison of this PCR protocol to results of quantitative PCR results with the same templates, described below, show that the detection limit for the standard *Korarchaeota* PCR was less than 10 copies per PCR reaction; however, the presence of PCR inhibitors in some DNA preparations, discussed above, reduced the effective detection limit in some or all of the samples.

### Molecular cloning, sorting, and sequencing

*Korarchaeota* PCR products were cloned using the TOPO T/A cloning kit (Invitrogen, Carlsbad, CA) according to manufacturer's protocol. DNA was extracted from 18 transformants from each *Korarchaeota*-positive sample (28 samples), except for 070714Y, which only had 13 transformants, and LHCcon2, which only had two. Each transformant was grown overnight, used for crude DNA lysis preparation [36], and screened by *Korarchaeota*-specific PCR. PCR products were phylotyped by RFLP by digesting separately with the restriction enzymes *Rsa*I and *Taq*I (Promega, Madison, WI). Restriction fragments were resolved by electrophoresis on a 2% agarose gel. At least one clone of each RFLP type from each spring was sequenced at Functional Biosciences, Inc (Madison, WI) using primers M13F and M13R. Five to 9 additional clones were randomly selected from each of 7 different clone libraries for sequencing to determine whether *Korarchaeota* phylotypes existed that were not resolved by the RFLP approach. None were identified. The Genbank accession numbers for 16S rRNA gene sequences generated in this study are JN573308 to JN573341.

### Phylogenetic analysis

Trimmed sequences with Phred 20 scores ≥600 bp were used to generate contigs with the EMBOSS application Merger [37]. Mismatches between forward and reverse reads were manually edited by referring to chromatograms. The EMBOSS application RevSeq was used to reverse complement the sequences oriented in the wrong direction [37]. Mallard [38] and Pintail [39] were used to check sequences for anomalies. Additional checks for chimeric artifacts were done with Bellerophon [40] and manually with BLASTn searches of sequence fragments from questionable sequences. No sequences were identified as likely chimeras. Sequences from this study and additional *Korarchaeota* sequences [28] were aligned using release 100 of the Silva database in ARB [41]. Sequences flagged as chimeric by others [28] were deleted. Analyses of the alignment were restricted to *E. coli* 16S rRNA gene nucleotide positions 264–1228, using the archaeal positional variability filter (pos_var_Archaea_100), with and without a 50% mask. The alignment was analyzed in ARB using neighbor-joining (Felsenstein correction), maximum parsimony, and maximum likelihood (AxML; Hasegawa-Kishino-Yano nucleotide substitution model). Bootstrap analyses (1000 replicates) for distance analysis and parsimony analyses were done in Phylip [42] using the programs seqboot, dnadist, and neighbor, and seqboot and dnapars, respectively, and consensus trees were built using consense.

### Quantitative Korarchaeota PCR

Quantitative real-time PCR (qPCR) was performed using an iCycler iQ Multicolor Real-Time PCR Detection System (BioRad, Hercules, CA, USA). Triplicate reactions contained 12.5 µl 2× PerfeCTa SYBR Green SuperMix for iQ (Quanta Biosciences, Gaithersburg, MD, USA), 2.5 µl template DNA and 400 nM of primers 236F [10] and Kor546r [10] in 25 µl total. Cycling conditions included an initial melting step of 95°C for 3 min followed by 50 cycles of 94°C for 15 s, 64°C for 15 s and 72°C for 45 s. Data collection using a SYBR-490 filter was enabled during the 72°C step for each cycle. Following amplification, melt curves for the products were generated by increasing temperature from 55°C to 95°C by 0.5°C increments for 10 s each. Ten-fold dilutions, ranging from 10^1^ to 10^7^ copies per reaction, of linearized plasmid containing the cloned *Korarchaeota* 16S gene SSW_L4_D06 [12] were used as a standard. Threshold cycles were calculated using the maximum correlation coefficient approach and data analysis was performed using version 3.1 of the iCycler iQ Optical System Software (BioRad), taking dilutions into account. In multiple qPCR runs, amplification efficiencies ranged from 89–95.5% and correlation coefficients for the standard curve ranged from 0.998 to 1.0. Due to the unique phylogenetic composition of hot spring microbiota, particularly in the GB [12], [36], it was exceedingly difficult to design “universal” primers for quantitative PCR. Also, due to the low biomass of many samples and high background absorbance, DNA yield could not routinely be accurately quantified. Therefore, qPCR results were normalized to sediment wet weight.

### Statistics relating Korarchaeota presence and abundance to physicochemical habitat

Non-metric multi-dimensional scaling (NMS) was used to explore relationships among geochemical analytes. NMS is an ordination technique well-suited to non-normal ecological datasets. It uses ranked distances and, thus, does not assume linear relationships. NMS employs an iterative process to reduce dimensionality of multivariate data by seeking a final configuration of *n* samples in *k*-dimensions that displays minimal stress [43]. Ordinations of dissolved analytes were conducted in PC-ORD (MjM Software Design) using autopilot mode and Sørensen (Bray-Curtis) distance measures. NMS analyses were completed for GB and YNP separately and for the composite data set. Each NMS consisted of 100 initial runs to identify the optimal number of axes. To allow for Monte Carlo testing, 50 runs used actual data and 50 runs used randomized data generated by PC-ORD. The final ordination was completed using 99 runs with the recommended number of axes. Ordinations of geochemical analytes were plotted with *Korarchaeota* presence and abundance to explore qualitative relationships between biotic and abiotic variables.

To test whether differences in variance among concentrations of individual analytes were significantly different in *Korarchaeota*-permissive and non-permissive samples (bulk water (Table S1) or particulate (Table 3, S3)), datasets were separated and analyzed using one-way ANOVA and independent samples t-tests. Since molar concentrations of some bulk water analytes spanned up to seven orders of magnitude, data were log-transformed. Two-sample Kolmogorov–Smirnov (K-S) tests were used to identify significant differences in analyte concentration distributions between *Korarchaeota*-optimal or sub-optimal (>10^4^ 16S rRNA gene copies g^−1^) versus marginal or non-permissive springs. K-S analyses were completed for the composite data set and separately for the GB and YNP data sets. Spearman's rho values, non-parametric correlation coefficients, were used to identify correlations between *Korarchaeota* abundance and bulk water geochemical data. Rho was subjected to a two-tailed t-test to determine statistical significance.

**Table 3:** Particulate geochemistry of selected springs and statistics relating analytes to *Korarchaeota* presence and abundance in selected Great Basin springs^a^.

	Carbon					Nitrogen	
	C_Total_ (wt. %)	C_Org_ (wt. %)	C_Inorg_ (wt. %)	δ^13^C_Total_ (‰)	δ^13^C_Org_ (‰)	N_Total_ (wt. %)	N_Org_ (wt. %)
Permissive (abundance)^b^							
GVS1 (O)	7.94±2.3	0.52±0.001	7.41	0.96±0.4	−23.26±0.06	0.04±0.001	0.04±0.001
HC1 (O)	7.73±2.4	0.27±0.02	7.46	0.80±0.2	−22.55±0.03	0.02	0.03±0.0001
LHC1 (O)	4.99±0.03	0.13	4.86	−1.71±0.4	−22.35	0.01±0.0001	0.01
LHC3 (O)	10.92±0.06	0.36±0.06	10.56	−1.49±0.1	−22.08±0.1	0.05	0.04±0.007
LHC4 (O)	10.28±0.03	0.36±0.01	9.92	−1.44±0.08	−22.55±0.02	0.03	0.04±0.006
SSWcon1 (O)	1.40±0.003	0.56	0.84	−8.63±0.2	−20.73	0.06±0.001	0.06
SVX2 (M)	0.33	0.31±0.001	0.03	−20.39	−21.66±0.06	0.02	0.03±0.001
Non-permissive							
GBS17A	0.59±0.004	0.56±0.07	0.02	−16.75±0.2	−18.71±0.5	0.04±0.0001	0.04±0.01
SV2	0.24	0.22±0.002	0.02	−20.59	−21.73±0.04	0.02	0.02±0.001
SVX3	0.21±0.004	0.16±0.001	0.05	−16.12±0.2	−21.04±0.04	0.02	0.02±0.0004
ANOVA tests for differences among abundance classes^b^							
p-value	0.027	0.895	0.029	<0.001*	0.149	0.786	0.649
T-tests for differences between permissive/non-permissive classes							
p-value	0.009	0.704	0.010	0.022	0.050	0.509	0.386

a Carbon and nitrogen content are expressed as weight percent (wt. %), C and N isotopic compositions are expressed in permil (‰) relative to PDB and air standards, respectively. C_Inorg_ (wt. %) was calculated by difference (C_Inorg_ = C_total_−C_org_). Most particulate geochemistry measurements were made in triplicate; error values are ±1 standard deviation (S.D.); the errors reflect sample heterogeneity and, thus, are sometimes larger than the analytical uncertainty for these measurements (uncertainties are generally, <0.2% for mass and ∼0.02‰ for isotopic compositions). Corresponding data for a limited number of YNP springs is in Table S3.

b Abundance is defined as O and M, which are “optimal”, >10^5^ cells/g and “marginal”, <10^4^ cells/g, respectively.

* Result was significant for this particular test when corrected for multiple hypotheses using the Bonferroni correction (β = 0.05; n = 7).

All ANOVA, K-S test, correlation, and t-test results were adjusted for the number of statistical tests performed by using the Šidák correction, which assumes that each analyte is independent [44]. Šidák corrections were calculated separately for bulk water and sediment particulate geochemical analytes and were applied except when a specific hypothesis relating a habitat parameter and *Korarchaeota* abundance was applied.

### Support vector statistics

A C-SVM model was developed to predict *Korarchaeota* presence and relative abundance using geochemical data. C-SVMs are powerful classification tools that have been applied to various problems in biology, including the prediction of protein behavior from primary sequence [45], [46], [47], improvement of disease diagnosis and prognosis [48], [49], and behavior of complex organic molecules in solution [50]. C-SVMs map two classes of training data to a higher dimensional space and subsequently find a maximally separating hyperplane between the two classes of vectors, which partitions the space [51]. This separation is strongly dependent on the choice of kernel function, a relationship between vectors of the form *K*(**x** *_i_*, **x** *_j_*), where **x** *_i_* is the vector of features from the *i* ^th^ sample (in this case an analyte) and *K* is a function relating two feature vectors from different data points (e.g., different springs) to a scalar value. We chose two functions, linear *K*(**x**_i_, **x**_j_) = **x** *_i_*•**x** *_j_* and radial basis *K*(**x**_i_, **x**_j_) = exp(−γ∥**x** *_i_*−**x** *_j_*∥^2^), γ>0, where γ is a dimensionless tuning parameter that determines when feature vectors are considered to be distant from one another and ultimately affects the trade-off between Type-I and Type-II error rates. These kernel functions were chosen because they are simple to implement and widely applicable to biological questions [45], [46], [47], [48], [49], [50]. A second dimensionless parameter, *C*>0, is used as a penalty score assessed against classifiers that place a training vector on the wrong side of the separating hyperplane. The choice of *C* determines the margin of the hyperplane, the distance between the closest feature vectors that are assigned to different categories, by allowing some individual training features to be misclassified. Both γ and C were determined empirically by cross-validation.

In this case, the two classes were samples in which *Korarchaeota* were present (“permissive”) or absent (“non-permissive”), as defined by qualitative PCR or “optimal/sub-optimal” (>10^4^ 16S rRNA gene copies g^−1^) or “marginal/non-permissive”, as defined by quantitative PCR. The space consisted of feature vectors **x** *_i_*, which consisted of all single analytes or all combinations of two analytes. Analytes were input as individual molar concentrations of individual analytes that were log-transformed and normalized from 0 to 1. Temperature data were normalized from 0 to 1 without log transformation. In addition, axes from NMS ordinations were tested as feature vectors of *Korarchaeota* abundance models.

C-SVMs were constructed in Java using the LIBSVM class library [52]. Training and evaluation were carried out using a 5-fold crossover model. Springs within the two categories were randomly divided into 100 sets (bootstraps) of training springs (80% of springs within each category) and evaluation springs (20% of springs within each category). Linear and radial basis SVMs were evaluated by a two-stage grid-search over their respective parameter spaces. The error penalty ‘C’ was allowed to range between 0 and 2500 with a granularity of 100 for the first stage, and 10 for the second. Similarly, the radial basis bias parameter gamma was allowed to range between 0 and 1 with granularity of 0.05 and 0.01, respectively, for the first and second stages of training. Preliminary accuracy, precision, and sensitivity measurements were estimated for each point in the parameter space using five-fold crossover validation with three replicate runs. The values of the parameters that gave the highest accuracy measurement were recorded.

On the basis of the initial survey, the abundance data sets and radial basis kernel were selected for more rigorous evaluation. Analytes that had not classified springs correctly with over 80% accuracy in either single analyte or two analyte classifiers were dropped from the final training sets to reduce the computational cost of additional bootstrap testing. These reduced datasets were subjected to the same analysis as previously, using the radial basis kernel function and 100 replicates to yield accuracy, precision, and sensitivity measurements for each classifier.

## Results and Discussion

### Korarchaeota diversity, distribution, and biogeography

DNA was successfully extracted from 99 of the 107 sediment samples as determined by PCR using primers specific for 16S rRNA genes of *Bacteria* and/or *Archaea*: 64 from YNP and 35 from the GB. Of those, *Korarchaeota* were detected in 15 YNP samples (23%) and 13 GB samples (37%), including a wide range of physicochemical, geological, and geographical settings and substrate types (e.g., fine and coarse sediments and photosynthetic mats; Table 1, 2, S1, S2). These included all 6 “thermal regions” and 6 of 8 “thermal areas” sampled in YNP (terminology following the Yellowstone Research Coordination Network [53]) and 4 of 5 thermal regions and 4 of 5 thermal areas in the GB. Notably, *Korarchaeota* were not detected in Sentinel Meadows in YNP, despite screening of 16 samples at that location. The only other thermal areas in which *Korarchaeota* were not detected were the White Creek Group in YNP and the Smith Creek area in GB, yet for each of these systems only a single sample was screened.

**Figure 1:**
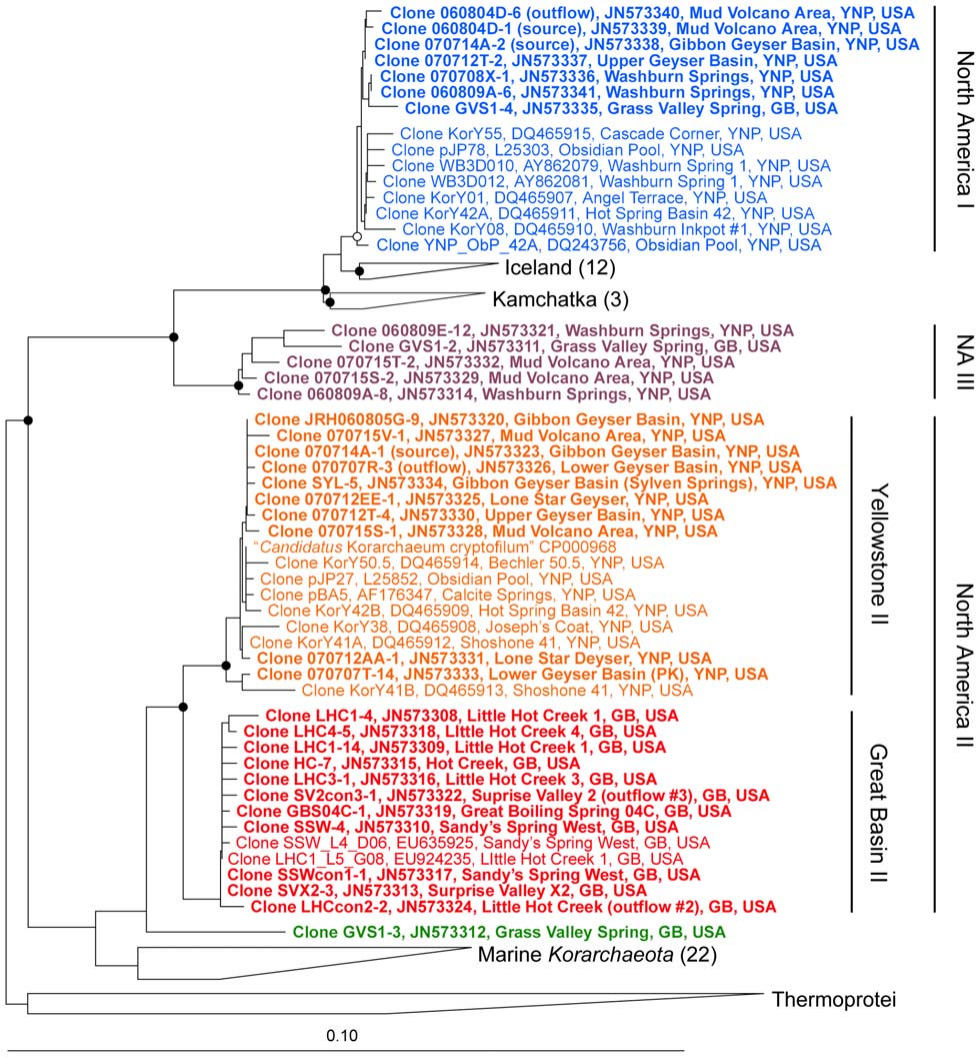
Distance tree with representative *Korarchaeota* 16S rRNA gene sequences created in ARB using *E. coli* nucleotide positions 264–1228. Sequences generated in this study are shown in bold for emphasis. (•) Major nodes supported by maximum likelihood, neighbor-joining and maximum parsimony trees. (°) Major nodes supported by 2 of 3 methods. Branching positions with or without a 50% mask were identical. Nodes receiving high (•) or moderate (°) support were also supported by bootstrap analysis (not shown). Monophyletic groups with sequences >98% from the same geographic location or habitat are collapsed with the number of sequences in the group indicated next to the wedge. For this analysis, redundant sequences (>99% identity) from the same sample were removed prior to analysis. Bar, 0.1 substitutions per nucleotide.

**Figure 2:**
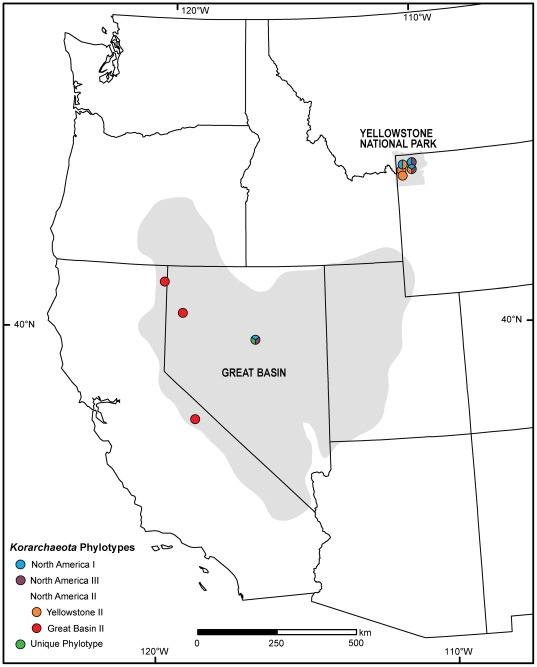
*Korarchaeota* phylotypes of the western U.S. mapped as colored circles. Split circles represent multiple phylotypes that occurred in one spring system. See Figure 1 for details on phylogenetic relationships.

Over 500 *Korarchaeota* 16S rRNA genes were screened by RFLP analysis and 90 genes were chosen for DNA sequencing. All 16S rRNA genes branched monophyletically within the *Korarchaeota* (Fig. 1). All but one comprised four phylogenetic clusters, which were non-randomly distributed with regard to geography (Fig. 1, 2). Two clusters belonged to the group designated “North America II” [28], closely related to clone pJP27 and “*Ca.* Korarchaeum cryptofilum” from Obsidian Pool [9]. One cluster, herein defined as “Yellowstone II”, was an exclusive inhabitant of YNP springs, with each member sharing >98% sequence identity to clone pJP27. The second, defined as “Great Basin II”, was an exclusive inhabitant of GB springs, each with 96–98% sequence identity to pJP27. “Great Basin II” was the only phylotype inhabiting springs along the western margin of the GB, yet it was not detected in Grass Valley Spring (GVS) in the central GB (Fig. 2). The monophyly of “Yellowstone II” was supported by neighbor-joining, maximum parsimony and maximum likelihood phylogenetic methods. The “Great Basin II” cluster was either monophyletic (Fig. 1) or branched basally to the “Yellowstone II” cluster.

A third cluster was nearly identical (>98% 16S rRNA gene identity) to clone pJP78 from Obsidian Pool, designated “North America I” [28]. It was comprised of YNP sequences and one sequence from GVS in the central GB (Fig. 1, 2). These sequences are related to monophyletic groups from hot springs in Iceland and Kamchatka [14], [16], [28]. The “North America I” group was monophyletic in all three phylogenetic methods, supporting the biogeographic structure reported by Reigstad et al. [28]. A fourth group, herein designated “North America III”, branched basally to the cluster including “North America I” and included sequences from YNP and GVS.

One sequence from GVS, GVS1–3, was unique and quite different from phylotypes described elsewhere (93–97% 16S rRNA gene identity). The phylogenetic position of GVS1–3 was inconsistent when analyzed by different phylogenetic approaches.

Phylotypes from marine hydrothermal sites were either a monophyletic sister group to a terrestrial lineage (Fig. 1) or formed several, deep branching lineages within the phylum. The latter structure is similar to what has been reported elsewhere [28]. This phylogenetic structure, along with the low phylogenetic diversity of terrestrial *Korarchaeota*, suggests a marine origin for *Korarchaeota* with subsequent niche-invasion to terrestrial geothermal habitats.

Each of the biogeographic phylotypes specific to Iceland, Kamchatka, and different regions of North America qualify as “geovars”, or geographic varieties [54]. Although it cannot be proven unequivocally that the geovars are absent from any particular location, it is notable that no geovar has been found by any research group outside of its reported geographic range [11], [12], [14], [16], [19], [28], [55]. The spatial distribution of these geovars is also intriguing, because YNP and GVS support greater phylogenetic diversity compared to sites in the western GB, and share genetic variants that have not been found elsewhere (Fig. 2). The higher diversity of *Korarchaeota* in YNP and GVS and the genetic similarity of some co-located phylotypes suggest that these springs share a historical connection, perhaps through the historical track of the mantle plume responsible for the Yellowstone Caldera. The low diversity of *Korarchaeota* in the western GB could be interpreted as a recent invasion of a single phylotype of *Korarchaeota* into these springs from refugia populations in YNP or in the central or eastern GB. Alternatively, *Korarchaeota* in the western GB may be genetically connected with populations further west. A broader geographic survey of *Korarchaeota* in the Western U.S. and a corresponding analysis of other thermophilic taxa may resolve these alternatives. Nevertheless, these data challenge the strict interpretation of Baas-Becking's dictum “*alles is overal:* maar *het milieu selecteert*” (everything is everywhere, but the environment selects)[56], [57] and is consistent with the proposal to elevate the interpretation of “*alles*” (everything) to the level of the bacterial or archaeal genus, at least in some cases [58].

### Temperature and pH of Korarchaeota habitats

The geochemical habitat supporting *Korarchaeota* populations was analyzed by relating geochemical measurements to both presence/absence data, as judged by PCR and quantitative population abundance data, as assessed by qPCR (Fig. S1).

**Figure 3:**
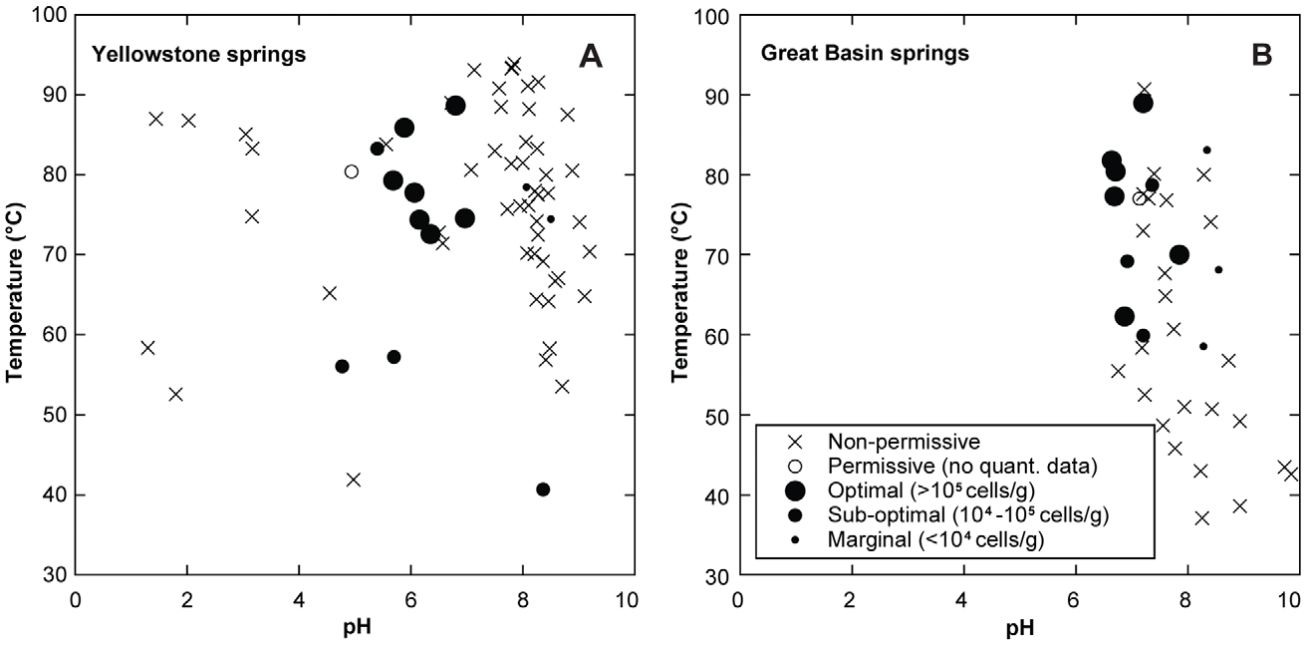
Temperature versus pH plots highlighting the results of quantitative PCR for *Korarchaeota* in samples from YNP (A) and the GB (B).

Samples in which *Korarchaeota* were detected ranged from 40.7–90.5°C with pH values from 4.78–7.85 (Fig. 3). However, the only *Korarchaeota*-positive sample below 55°C (JRH060805G; 40.7°C) was in the outflow system of 070714A, whose high-temperature source was also positive. In most outflow systems sampled, *Korarchaeota* were abundant at or near the source pool with less robust populations as temperatures decreased in outflow channels. This was most clearly illustrated in outflow systems of Little Hot Creek (LHC) and Sandy's Spring West (SSW) in the GB (Fig. S1, light grey bars, Fig. S2). LHC is formed by the confluence of three 78.7–82°C surface expressions, LHC-1, LHC-3, and LHC-4 [55]. *Korarchaeota* abundance in samples from all three sources was among the highest observed in the study (0.3–2.0×10^6^ gene copies g^−1^; Fig. S1). Yet, the *Korarchaeota* population declined in successive samples along the outflow channel at 69.2 and 59.9°C and was below the PCR detection limit at 51.0, 43.0, and 37.1°C (Fig. S2). In SSW, the 86.6°C source contained a modest population of *Korarchaeota* (1.3×10^4^ gene copies g^−1^), which increased 15-fold in an outflow sample at 70°C, then decreased to near the qPCR detection limit at 58.6°C, and was undetectable at 50.7°C (Fig. S1). In the GB, a marginally significant, positive relationship was observed between *Korarchaeota* abundance and temperature (rho = 0.406, p = 0.076, n = 20) further showing that the prime habitat of *Korarchaeota* is at or near high temperature spring sources. However, it should be noted that *Korarchaeota* populations were also detected in several 55–65°C non-flowing springs in both the GB and YNP (GBS04C, 62.3°C; 060804D; 070715T, 57.3°C) suggesting *Korarchaeota* can also grow and compete at these lower temperatures. Other studies have detected *Korarchaeota* in six 55–65°C springs in YNP and in Kamchatka [19]. In all, our study suggests a habitat temperature range of 55 to >90.5°C for these terrestrial *Korarchaeota* lineages, which is in agreement with the habitat surveys of others [11], [28], cultivation studies of “*Ca.* K. cryptofilum” [9], [10], and spans a temperature range typical of cardinal growth temperatures of *Bacteria* and *Archaea* [59].

*Korarchaeota* showed a strong preference for neutral to mildly acidic springs in both YNP and the GB (Fig. 3). K-S tests indicated that pH values, or H^+^ activity (pH = −log(*a*H+), as measured by a field pH meter), differed significantly between *Korarchaeota*-optimal/sub-optimal and marginal/non-permissive samples when GB and YNP were analyzed separately and together (Fig. S3). In addition, Spearman's rank correlation coefficients showed that *a*H^+^ positively correlated with *Korarchaeota* abundance within YNP and within the combined dataset (Fig. S4).

In YNP, *Korarchaeota* were detected in 0% of low pH samples (pH<4.7), 75% of mildly acidic samples (pH 4.7–7), and 7% of high pH samples (pH>7). Similarly, when abundance was considered, all YNP springs with *Korarchaeota* populations >10^5^ cells g^−1^, herein defined as “optimal”, were between pH 5.7 and 7.0, whereas no “optimal” habitats were outside that pH range. Although relatively few samples of pH<4.7 were screened, the negative PCR results obtained here are consistent with the apparent absence of *Korarchaeota* in nine springs with pH<4.25 in Iceland and Kamchatka [11], [19]. In all, the pH range for YNP *Korarchaeota* described here is similar to that reported previously in YNP (5.6–6.6 [11]), Iceland (5.0–6.5 [28]), and Kamchatka (5.5–6.5 [19], [28]) and in agreement with the pH of the medium used to cultivate “*Ca.* K. cryptofilum” (6.5 [9]).

**Figure 4:**
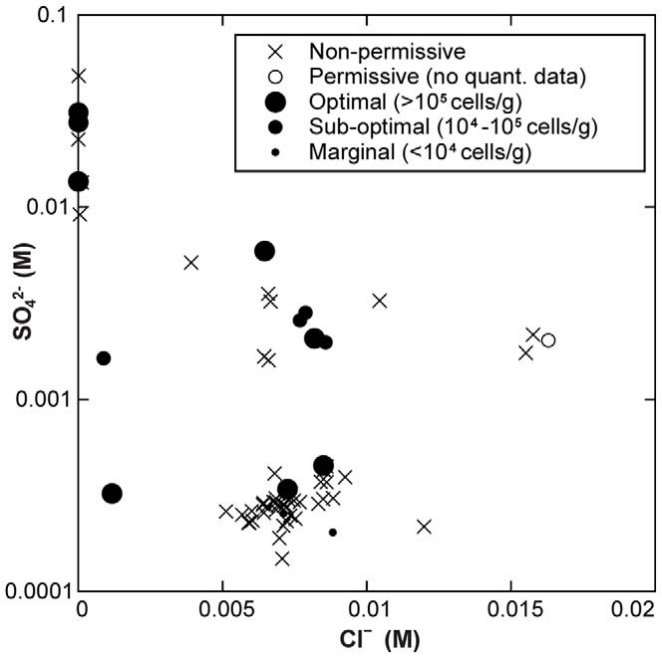
Chloride versus sulfate plot for YNP highlighting higher incidence and abundance of *Korarchaeota* in vapor-influenced springs (sulfate >1 mM [**60**]) in waters with (chloride 5–10 mM) or without (low chloride) input of deeply-sourced hydrothermal water. Springs of higher chloride concentration likely represent the liquid-water system of deep hydrothermal sources subjected to subsurface boiling [60].

In Yellowstone, many acidic springs are formed by vapor condensate [60]. These springs are acidified to various degrees by sulfuric acid that originates from H_2_S that partitions into the vapor phase during adiabatic decompression, concentrates as vapor condenses at or near the surface, and is subsequently oxidized biotically and abiotically to sulfuric acid. The sulfuric acid system buffers within a pH range of ∼1.5 to ∼3.5, explaining the abundance of YNP springs in this pH range [61]. To determine the importance of this process in *Korarchaeota* habitability in YNP, we examined the relationship between *Korarchaeota* and sulfate concentration. We observed a much higher incidence of *Korarchaeota* in YNP springs with sulfate concentrations over 1 mM, the proposed upper estimate for the sulfate concentration in the YNP deep geothermal reservoir [60] (Fig. 4; χ^2^ p = 0.016, df = 1), and a positive correlation between *Korarchaeota* abundance and sulfate concentration in YNP springs (Fig. S4; rho = 0.575, p = 0.006, n = 21). However, since *Korarchaeota* exclusively populate hot springs outside of the pH range of the sulfuric acid buffering system, *Korarchaeota*-permissive springs are evidently influenced by other water sources. Thus, we term sulfate-rich springs that are conducive to *Korarchaeota* “vapor-influenced” to distinguish them from “vapor-dominated” springs that are sourced mainly or exclusively by vapor condensate and whose pH is controlled by sulfuric acid. It is also noteworthy that a few YNP springs with low sulfate were “optimal” for *Korarchaeota* (070715S, 070712AA, and 070707T), illustrating that vapor influence is not required for *Korarchaeota*. Slightly acidic pH in these springs might be generated by enrichment with CO_2_ as spring fluid rises to the surface, by input of oxidized surface waters, or by fluid interactions with soil. The highly variable chloride concentration in *Korarchaeota*-permissive springs (0.27–578 mg L^−1^) shows that *Korarchaeota* can, but do not exclusively, inhabit springs fed by waters of deep hydrothermal origin (Fig. 4); however, *Korarchaeota* were most abundant in springs with low Na^+^ (Fig. S4; rho = −0.533, p = 0.013, n = 21), again suggesting that springs with significant inputs of vapor condensate or meteoric water are more likely to be preferred habitats.

Vapor-influenced features are characteristic of the Greater Obsidian Pool Area, Sylvan Springs, and Washburn Hot Springs [13], [60]. It is noteworthy that the ten *Korarchaeota*-permissive springs in these three “thermal areas” were all higher in pH than the nine co-localized non-permissive springs. Conversely, in the River Group in the Lower Geyser Basin, which is generally regarded as liquid water-dominated [60], *Korarchaeota* were found in the lowest pH sample taken, (070707T). These data demonstrate that moderately acidic pH is correlated with *Korarchaeota* habitability, irrespective of geographic location.

A relationship between *Korarchaeota* and pH was less evident from presence/absence data alone in GB samples (Fig. 3B). For example, when data from springs >55°C were equally partitioned into high and low pH categories, no difference between the two categories was observed (χ^2^ p = 0.43, df = 1). However, springs with pH<7.2 had higher *Korarchaeota* abundance (mean 1.43×10^6^ gene copies g^−1^; n = 7) than those with pH>7.2 (mean 4.32×10^4^ gene copies g^−1^; n = 7). Parametric ANOVAs indicated differences in mean pH values that were marginally statistically significant (p = 0.077). Nevertheless, K-S tests showed that the distribution of *a*H^+^ values differed significantly between *Korarchaeota*-optimal/sub-optimal and marginal/non-permissive samples (Fig. S3). GB springs are generally regarded as liquid water-dominated systems and pH ranges are correspondingly narrow [62], which may account for the subtle differences in mean pH observed between *Korarchaeota*-optimal/sub-optimal and marginal/non-permissive samples. It is also noteworthy that pH and temperature are auto-correlated in GB springs because of CO_2_ removal due to degassing and autotrophy in hot spring outflow channels; thus, based on these data alone, it is impossible to disentangle the effects of pH and temperature on *Korarchaeota* populations in GB springs.

The low incidence of each of the four phylogenetic clusters of *Korarchaeota* precluded robust statistical analysis of the phylogenetic groups separately; however, they appeared to inhabit similar springs and often cohabitated (Table 1, 2; Fig. 2). For example, GVS hosted each major clade found in North America except NA II, as well as phylotype GVS1–3, which was unique to this spring.

### Bulk water geochemistry of Korarchaeota habitats

A variety of statistical tests were applied to determine whether other geochemical measurements correlated with *Korarchaeota* abundance. K-S tests indicated significant differences in the distribution of individual analyte concentrations between *Korarchaeota*-optimal/sub-optimal and marginal/non-permissive samples (p<0.05; Fig. S3), but only *a*H^+^ and thallium (Tl) showed significant differences after Šidák corrections. Spearman's rho non-parametric correlation coefficients indicated several significant relationships after Šidák corrections (Fig. S4). In YNP, ions diagnostic of water that has undergone extensive water-rock reaction, such as Na^+^, negatively correlated with *Korarchaeota* abundance, whereas and *a*H^+^ and SO_4_^2−^, both diagnostic of vapor influence, positively correlated with *Korarchaeota* abundance (Fig. S4). In contrast, SO_4_^2−^ was negatively correlated with *Korarchaeota* abundance in GB springs, demonstrating that mildly acidic pH and not SO_4_^2−^, *per se*, is correlated with *Korarchaeota* abundance (Fig. S4). In all locations, *Korarchaeota* abundance was positively associated with metals such as Mn, Fe, Cr, and Mg (Fig. S3, S4), but these relationships are likely functions of pH. These metal ions become increasingly soluble with decreasing pH [63], [64], [65], particularly within the optimal pH range for *Korarchaeota*. Similarly, the negative relationship between *Korarchaeota* and ions such as Mo and Sb may be due to their low solubility at low pH [64], [65]. Spearman's rho showed that Tl concentrations in YNP samples negatively correlated with *a*H^+^ and *Korarchaeota* (Fig. S4). These biogeochemical relationships were further illustrated by NMS plots.

Non-metric multi-dimensional scaling highlighted the geochemical dissimilarity between GB and YNP hot springs (Fig. S5) and the distinctness of thermal regions, particularly within the GB (Fig. S6, S7). NMS plots based on geochemical data from both YNP (Fig. S6) and GB (Fig. S7) showed strong positive correlations between *a*H^+^ and metals known to have increased concentrations at low pH and negative correlations between *a*H^+^ and metals that are less abundant at low pH. In YNP, many springs defined as “optimal” for *Korarchaeota* are a few degrees off the trajectory of the *a*H^+^ vector, again suggesting that springs with some influence from H_2_S enrichment and oxidation are preferred habitats, whereas highly acidic end-members directly on the trajectory of the *a*H^+^ vector are not permissive for *Korarchaeota*. This is most clearly illustrated for springs in the Washburn Hot Spring Area (Fig. S6). In contrast, in the GB, the LHC system is directly on the trajectory of the *a*H^+^ vector. However, the spread of *Korarchaeota*-permissive springs in the YNP, GB, and all-system NMS plots underscore the extremely wide geochemical diversity of *Korarchaeota* habitats and likely support a model where mildly acidic pH is a driving factor in *Korarchaeota* abundance, regardless of the mechanisms maintaining that pH.

### Particulate geochemistry of Korarchaeota habitats

**Figure 5:**
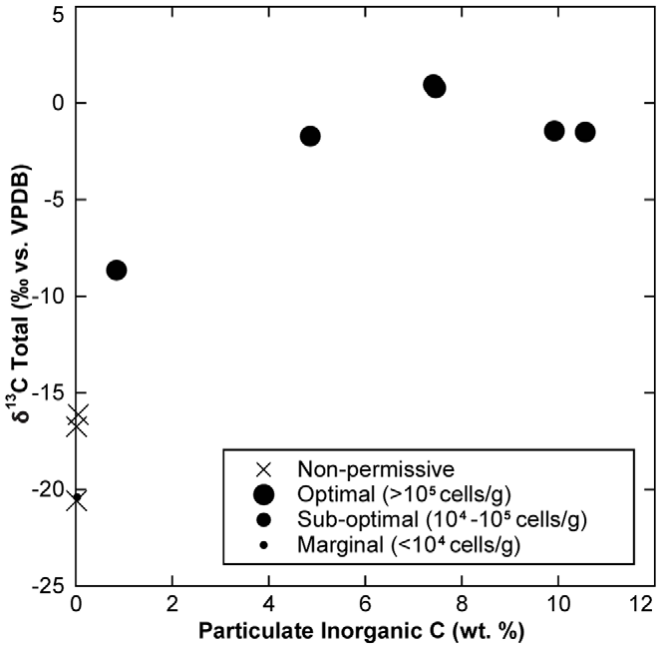
Inorganic carbon content versus δ^13^C_Total_ for sediment particulate material collected in selected Great Basin springs highlighting higher incidence and abundance of *Korarchaeota* in springs that are actively precipitating carbonate, as indicated by high inorganic C content and heavy δ^13^C_Total_ values.

Since others have noted strong correlations between solid-phase geochemistry and hot sediment microbiota [4], sediment particulate C and N geochemistry were measured in a number of sediment samples. In the GB, strong relationships were noted between *Korarchaeota* abundance and particulate total carbon and inorganic carbon (Table 3; Fig. 5). This is consistent with high carbonate alkalinity measured in the bulk water of many *Korarchaeota*-permissive GB springs (Fig. S4, S7). Of all springs sampled, Little Hot Creek, Hot Creek, and Grass Valley Spring had the highest *Korarchaeota* abundance and the highest alkalinity. δ^13^C_Total_ levels were significantly heavier within these springs, which is consistent with a magmatic source of CO_2_ [66]. The positive relationship between carbonate content and *Korarchaeota* abundance in GB springs may be due to buffering by the carbonic acid system, which maintains optimal pH, effectively substituting for the vapor condensation-driven mild acidification of many YNP springs. Surprisingly, although *Korarchaeota* are predicted to be peptide fermenters and to rely on microbial co-inhabitants to produce essential vitamins, cofactors, and purines [9], no relationship between *Korarchaeota* presence or abundance and organic carbon or nitrogen content was observed (Table 3). Although relatively few YNP samples were analyzed for particulate geochemistry (Table S3), all sediments had extremely low carbonate content.

### A Korarchaeota classification support vector machine

Due to the limited power of statistical tests relating presence/absence data with such a large number of geochemical measurements, and to account for possible complex relationships between analytes and habitability, we applied a C-SVM in an attempt to create a model that determines *Korarchaeota* habitability based on bulk water geochemistry data. For datasets comprised only of YNP samples, pH alone or pH in combination with another analyte performed among the best of all models derived from single analyte or two analyte combinations (Table 4), consistent with the interpretations described above. In the GB, models based on pH were also predictive; however, models based on K^+^ or K^+^ combined with pH or alkalinity were better, reaching an accuracy of 95%. In the GB, K^+^ varied over roughly an order of magnitude, with *Korarchaeota* inhabiting springs of intermediate K^+^. We do not infer that K^+^ causes *Korarchaeota* habitability. Instead, it might be particularly diagnostic for the unique chemistry of the three main geothermal areas that were tested in the GB (Fig. S7), which either host robust *Korarchaeota* populations at permissive temperatures (e.g., Hot Creek/LHC systems) or tend not to host robust *Korarchaeota* populations (e.g., GBS and SV systems). Thus, it is unclear whether models based on K^+^ can be extrapolated to other systems in the GB or outside the GB. Classifiers using temperature either alone or in combination with pH did not perform well in either geographical region. Classifiers based on NMS axes performed similarly to one- and two-analyte classifiers, with accuracies ranging from 68–91%. Some highly predictive analytes, such as K^+^ and carbonate alkalinity, strongly correlated to NMS axes (Fig. S5, S6, S7), which further suggests some underlying structure within geochemical data corresponded to *Korarchaeota*.

**Table 4:** Results of ecological niche modeling using a C-SVM based on *Korarchaeota* abundance and bulk geochemistry data.

		5-fold Crossover Analysis (n = 100)			Cross-system Compare			Parameters	
Training Set	Best Analytes^a^	Accuracy^b^	Precision^c^	Sensitivity^d^	Accuracy	Precision	Sensitivity	γ	C
YNP	pH/Alk.	**0.89**	0.90	0.81	0.83	0.43	1.0	0.12	1481
	pH	0.84	0.98	0.68	0.87	0.87	0.71	0.22	1971
	pH/K+	0.77	0.48	0.75	0.83	0.57	0.80	1.05	2501
	pH/Temp	0.77	0.60	0.67	**0.91**	0.71	1.0	0.31	461
	1°+2° Axes	0.77	0.39	0.77	0.55	0.17	0.17	0.05	401
	1° Axis	0.68	0.00	0.00	0.73	0.00	NA	0.05	1
	Temp	0.67	0.00	0.00	0.70	0.70	0.0	0.07	2411
GB	pH/K+	**0.95**	0.85	0.98	0.58	0.64	0.41	0.40	81
	K+/NO_3_	**0.95**	0.85	0.98	0.67	0.00	NA^e^	1.01	1671
	K+	0.94	0.81	0.98	0.48	0.36	0.29	0.16	371
	1°+2° Axes	0.91	0.67	1.0	0.68	0.30	0.50	0.30	1
	1° Axis	0.87	0.52	1.0	0.65	0.30	0.43	0.05	721
	pH	0.87	0.77	0.80	**0.70**	1.0	0.52	0.48	1581
	pH/Temp	0.76	0.36	0.71	0.67	1.0	0.50	0.06	801
	Temp	0.70	0.03	0.51	0.67	0.00	NA	0.05	1
ALL	pH/Alk.	**0.88**	0.82	0.91				0.40	2101
	pH	0.85	0.83	0.74				0.24	1851
	1°+3° Axes	0.84	0.50	0.95				0.16	211
	pH/K+	0.83	0.60	0.83				0.24	2051
	3° Axis	0.82	0.50	0.86				0.12	21
	pH/Temp	0.81	0.60	0.75				0.85	101
	Alk.	0.78	0.44	0.80				0.46	151
	Temp	0.68	0.00	0.17				0.92	1711

a SVMs were created using a radial basis kernel for all single and two analyte combinations. Analytes and analyte combinations that had not appeared in any classifier scoring over 80% accuracy with 5 bootstraps were dropped from the final training sets to reduce the computational burden of additional testing. These reduced data sets were subjected to the same analysis as previously, using the radial basis kernel function and 100 replicates to yield accuracy, precision, and sensitivity measurements for each classifier. Models were ranked by accuracy and the most accurate classifiers are shown with the results of pH and Temperature-based classifiers for comparison.

b Accuracy = [tp+tn]/ [tp+tn+fp+fn], where tp is true positives, tn is true negatives, fp is false positives, and fn is false negatives.

c Precision = tp/[tp+fp].

d Sensitivity = tp/[tp+fn] (sometimes termed ‘Recall’).

e NA indicates that the precision or sensitivity cannot be calculated due to the absence of positive calls.

C-SVMs trained on data from one geographic region could not generally be applied to the other geographic region due to differences in the kernel function (γ) and the penalty parameter (C), both of which are highly dependent on the training data. YNP and GB geothermal springs are in dramatically different geological settings, giving rise to springs with distinct geochemistry, as illustrated by NMS plots (Fig. S5). It is therefore not surprising that models derived from one system are generally not applicable to the other system. To attempt to create a model that could predict *Korarchaeota* habitability outside of YNP and the GB, models based on a combined dataset were created and evaluated. pH and pH combined with carbonate alkalinity, K^+^, or temperature were the most predictive with the combined dataset, underscoring the importance of pH in defining *Korarchaeota* habitability. The primary and tertiary axes from the combined NMS model were also predictive. GB and YNP separate predominately along the primary axis (Fig. S5). This axis shows analytes characteristic of water that has undergone extensive water-rock interaction, such as Na^+^ and Cl^−^, decrease as analytes characteristic of vapor-influenced springs, such as *a*H^+^, Cr, and Fe, increase. Along the tertiary axis, analytes such as As, Sb, and Mo increase with decreasing SO_4_^2−^, Mn, K, Ca^2+^. C-SVM models should be applied to new geothermal systems in order to evaluate and refine them.

### Conclusions

We present a census of *Korarchaeota* phylogenetic diversity and geochemical habitat in YNP and GB hot springs. In agreement with other studies, there is clear biogeographic structure among *Korarchaeota* populations with very limited phylogenetic diversity. Endemism among terrestrial *Korarchaeota* demonstrated here and elsewhere [19] adds to a growing body of literature of species- to strain-level endemism among obligate thermophiles [67]. Whether endemism among thermophiles affects ecological functioning and whether endemism exists at higher taxonomic levels of thermophiles remains unanswered. The low diversity and shallow branching of terrestrial *Korarchaeota* is consistent with a marine origin for *Korarchaeota* with subsequent colonization of terrestrial geothermal habitats. The study revealed that *Korarchaeota* strongly prefer neutral to mildly acidic springs. In YNP most of these springs are “vapor-influenced” springs that are partially sourced by vapor condensate that has been acidified due to oxidation of sulfide to sulfuric acid. Yet, *Korarchaeota* do not inhabit acid-sulfate springs whose pH is dominated by the sulfuric acid buffering system, suggesting that neutralization by mixing with deeply-sourced geothermal water or meteoric water that has some buffering capacity, possibly by interaction with soils and sediments, is necessary for robust *Korarchaeota* populations. In YNP, 75% of sampled springs in the range of pH 4.7–7.0 supported *Korarchaeota* populations, suggesting that pH, alone, is an important predictor of *Korarchaeota* habitability. In the GB, high temperature sources actively precipitating carbonate with pH<7.2 are preferred habitats. The mildly acidic pH of these systems is likely controlled by the carbonic acid buffering system. The neutral to moderately acidic pH of preferred *Korarchaeota* habitats is consistent with the proposed metabolism of “*Ca.* Korarchaeum cryptofilum”, peptide fermentation coupled with proton reduction to H_2_ [9]. Both substrates, protons and dissolved organic carbon, are enriched with acidity. However, the ecological niche of *Korarchaeota* is not exclusively driven by increased proton availability because they do not typically inhabit vapor-dominated acidic end-members. C-SVMs based on pH, or pH along with another analyte, provided highly accurate ecological niche models for *Korarchaeota* in YNP, the GB, or the combined dataset. Models trained on data from combined YNP and GB datasets provide the best possible models for predicting *Korarchaeota* niches in unsampled spring systems, although extrapolation of these models to other geothermal systems should be evaluated critically. We also acknowledge the potential limitations of using bulk water geochemistry to predict sediment microbiology and advocate analyses of solid-phase geochemistry and pore water chemistry to improve future studies.

## Supporting Information

Figure S1 **Quantitative real-time PCR results showing the concentration of** ***Korarchaeota*** **16S rRNA genes in representative permissive sediments.** Light grey bars indicate outflow sets. ^a^Boulder OF3 and SSW con2 contained less than 10 copies per qPCR tube, which were extrapolated from the standard curve and may be below the reliable detection limit. Error bars indicate standard deviation (n = 3).
(PDF)

Figure S2 ***Korarchaeota*** **abundance, as determined by qPCR, decreased with decreasing temperature along the Little Hot Creek outflow system (GB).** Isotherms were modeled using point temperature data from the sample sites shown here. The only channels delineated are those for which abundance data were available.
(PDF)

Figure S3 **Two-sample Kolmogorov–Smirnov (K-S) tests indicated significant differences in analyte concentrations between** ***Korarchaeota*** **-optimal/sub-optimal (>10^4^ 16S rRNA gene copies g^−1^) and marginal/non-permissive samples.** These analyses were completed for the composite data set and separately for the GB and YNP data sets. K-S results are listed from most to least significant. Only results significant at the 0.05 level are shown (dark gray bars). Light gray bars indicate significant results under Šidák corrections. H^+^ was determined from field pH measurements and reflects the activity of H^+^ (*a*H^+^) and not concentration.
(PDF)

Figure S4 **Non-parametric correlation coefficients, or Spearman's rho values, indicated correlations between** ***Korarchaeota*** **abundance and individual geochemical analytes.** These analyses were completed for the composite data set and separately for the GB and YNP data sets. Only results significant at the 0.05 level are shown (dark gray bars). Light gray bars indicate significant results under Šidák corrections. H^+^ was determined from field pH measurements and reflects the activity of H^+^ (*a*H^+^) and not concentration.
(PDF)

Figure S5 **An NMS plot shows relationships among multiple geochemical variables from all YNP and GB sites.** The ordination of geochemical analytes from all sites yielded a reliable, three-axis solution (stress = 9.499; p = 0.0196; cumulative r^2^ = 0.941). Axes 1 and 3 are shown because they best illustrated the relationships between geochemistry and *Korarachaeota* abundance and the geochemical dissimilarity between YNP and GB. Distance between sample sites is proportional to dissimilarity in geochemical composition. Geochemically similar sites cluster closely together, as shown by the separation of many YNP from GB sites. Vectors in black illustrate correlations of individual analytes to ordination axes and are directed toward samples in which those analytes are elevated. The magnitude of these relationships is indicated by the length of the vectors, with the longest lines corresponding to the strongest relationships. Only r^2^≥0.2 are shown. H^+^ was determined from field pH measurements and reflects the activity of H^+^ (*a*H^+^) and not concentration.
(PDF)

Figure S6 **An NMS plot shows relationships among multiple geochemical variables from YNP sites.** The ordination of geochemical analytes from the YNP samples yielded a reliable, two-axis solution (stress = 7.272; p = 0.0196; cumulative r^2^ = 0.975). Distance between sample sites is proportional to dissimilarity in geochemical composition. Geochemically similar sites cluster closely together, as shown by the separation of sites from different geothermal regions. Vectors in black illustrate correlations of individual analytes to ordination axes and are directed toward samples in which those analytes are elevated. The magnitude of these relationships is indicated by the length of the vectors, with the longest lines corresponding to the strongest relationships. Only r^2^≥0.2 are shown. H^+^ was determined from field pH measurements and reflects the activity of H^+^ (*a*H^+^) and not concentration.
(PDF)

Figure S7 **An NMS plot shows relationships among multiple geochemical variables from GB sites.** The ordination of geochemical analytes from the GB samples yielded a reliable, two-axis solution (stress = 5.176; p = 0.0196; cumulative r^2^ = 0.903). Distance between sample sites is proportional to dissimilarity in geochemical composition. Geochemically similar sites cluster closely together, as shown by the separation of sites from different geothermal regions. Vectors in black illustrate correlations of individual analytes to ordination axes and are directed toward samples in which those analytes are elevated. The magnitude of these relationships is indicated by the length of the vectors, with the longest lines corresponding to the strongest relationships. Only r^2^≥0.2 are shown. H^+^ was determined from field pH measurements and reflects the activity of H^+^ (*a*H^+^) and not concentration.
(PDF)

Table S1 **Physicochemical measurements in hot spring bulk water.**
(XLSX)

Table S2 **Description of GB and YNP hot springs in which** ***Korarchaeota*** **16S rRNA genes were not detected.**
(DOC)

Table S3 **Particulate geochemistry of selected springs and summary of statistics relating analytes to** ***Korarchaeota*** **presence and abundance in selected Yellowstone springs.**
(DOCX)
