# Supplementary figures and images for: Korarchaeota Diversity, Biogeography, and Abundance in Yellowstone and Great Basin Hot Springs and Ecological Niche Modeling Based on Machine Learning
Source: PLoS One. 2012 May 4;7(5):e35964. doi: 10.1371/journal.pone.0035964 (PMC3344838; doi:10.1371/journal.pone.0035964)

Korarchaeota 16S rRNA gene copies  
per gram sediment (wet weight)

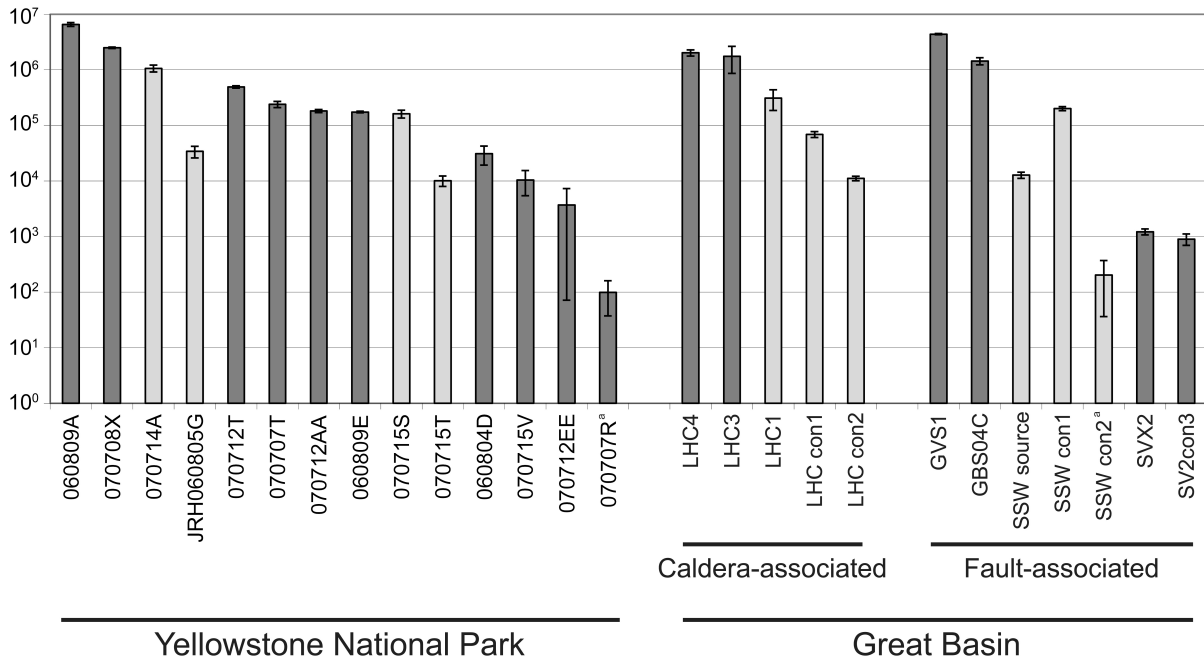

Supplement: Figure S1 — Quantitative real-time PCR results showing the concentration of Korarchaeota 16S rRNA genes in representative permissive sediments. Light grey bars indicate outflow sets. aBoulder OF3 and SSW con2 contained less than 10 copies per qPCR tube, which were extrapolated from the standard curve and may be below the reliable detection limit. Error bars indicate standard deviation (n = 3). (PDF) [file pone.0035964.s001.pdf]

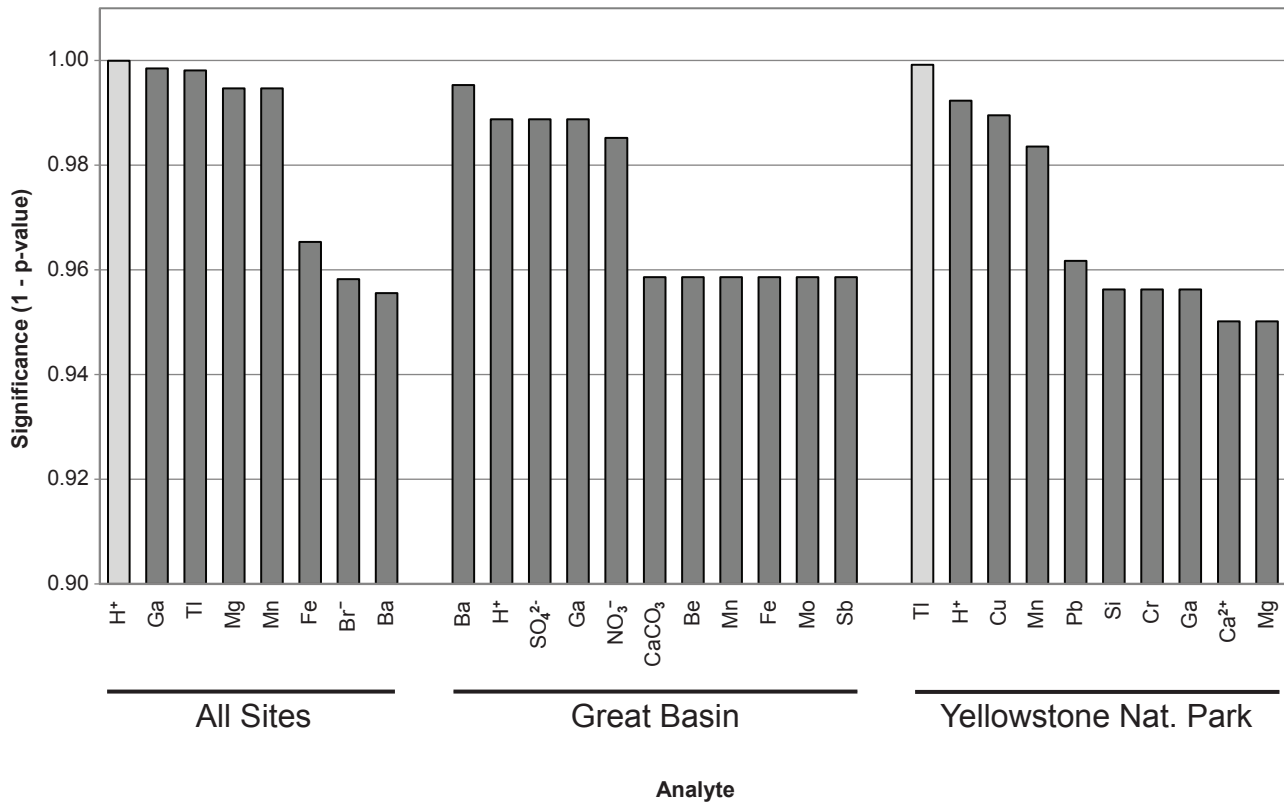

Supplement: Figure S3 — Two-sample Kolmogorov–Smirnov (K-S) tests indicated significant differences in analyte concentrations between Korarchaeota -optimal/sub-optimal (>104 16S rRNA gene copies g−1) and marginal/non-permissive samples. These analyses were completed for the composite data set and separately for the GB and YNP data sets. K-S results are listed from most to least significant. Only results significant at the 0.05 level are shown (dark gray bars). Light gray bars indicate significant results under Šidák corrections. H+ was determined from field pH measurements and reflects the activity of H+ (aH+) and not concentration. (PDF) [file pone.0035964.s003.pdf]

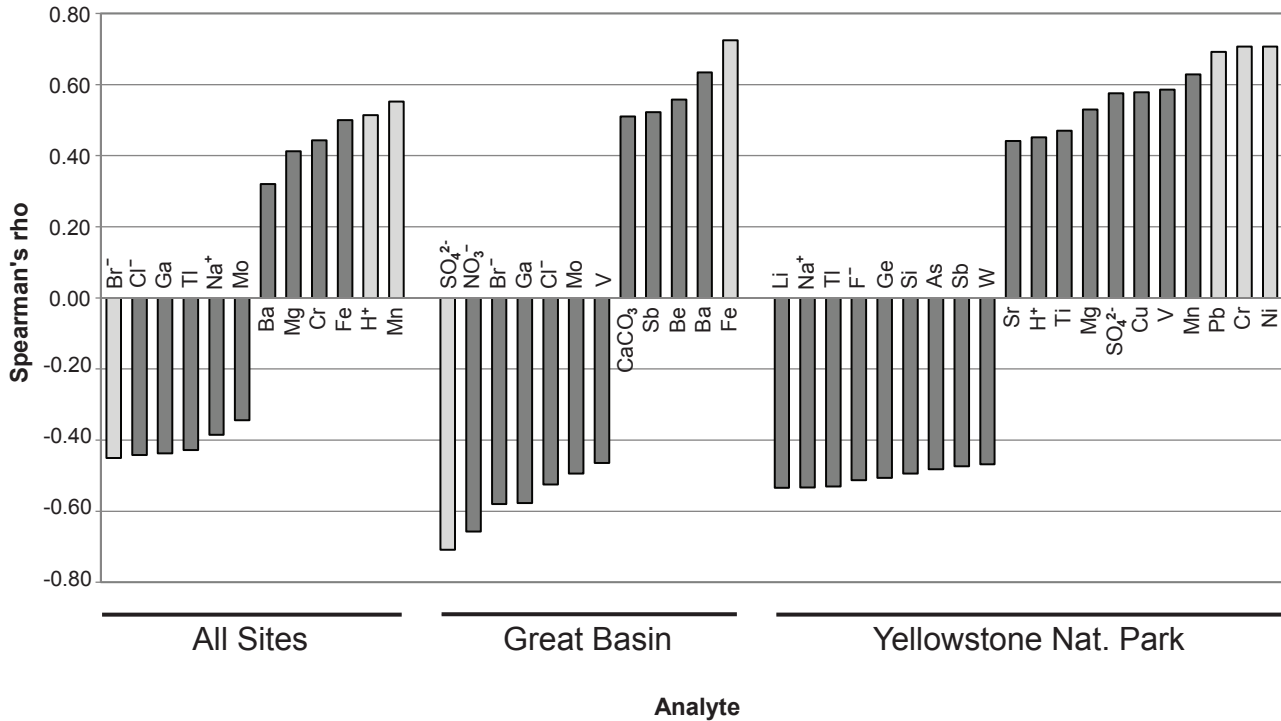

Supplement: Figure S4 — Non-parametric correlation coefficients, or Spearman's rho values, indicated correlations between Korarchaeota abundance and individual geochemical analytes. These analyses were completed for the composite data set and separately for the GB and YNP data sets. Only results significant at the 0.05 level are shown (dark gray bars). Light gray bars indicate significant results under Šidák corrections. H+ was determined from field pH measurements and reflects the activity of H+ (aH+) and not concentration. (PDF) [file pone.0035964.s004.pdf]

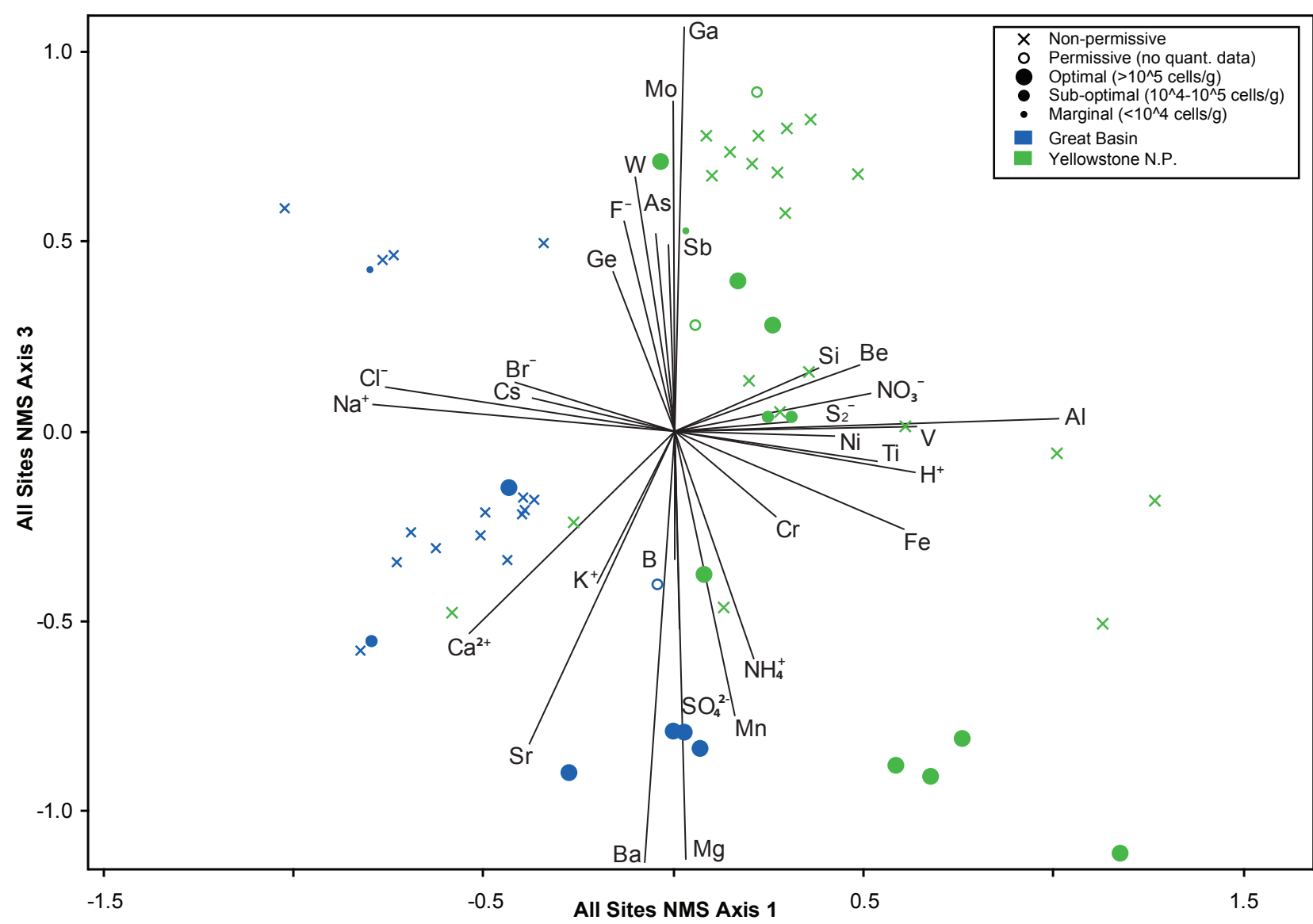

Supplement: Figure S5 — An NMS plot shows relationships among multiple geochemical variables from all YNP and GB sites. The ordination of geochemical analytes from all sites yielded a reliable, three-axis solution (stress = 9.499; p = 0.0196; cumulative r2 = 0.941). Axes 1 and 3 are shown because they best illustrated the relationships between geochemistry and Korarachaeota abundance and the geochemical dissimilarity between YNP and GB. Distance between sample sites is proportional to dissimilarity in geochemical composition. Geochemically similar sites cluster closely together, as shown by the separation of many YNP from GB sites. Vectors in black illustrate correlations of individual analytes to ordination axes and are directed toward samples in which those analytes are elevated. The magnitude of these relationships is indicated by the length of the vectors, with the longest lines corresponding to the strongest relationships. Only r2≥0.2 are shown. H+ was determined from field pH measurements and reflects the activity of H+ (aH+) and not concentration. (PDF) [file pone.0035964.s005.pdf]

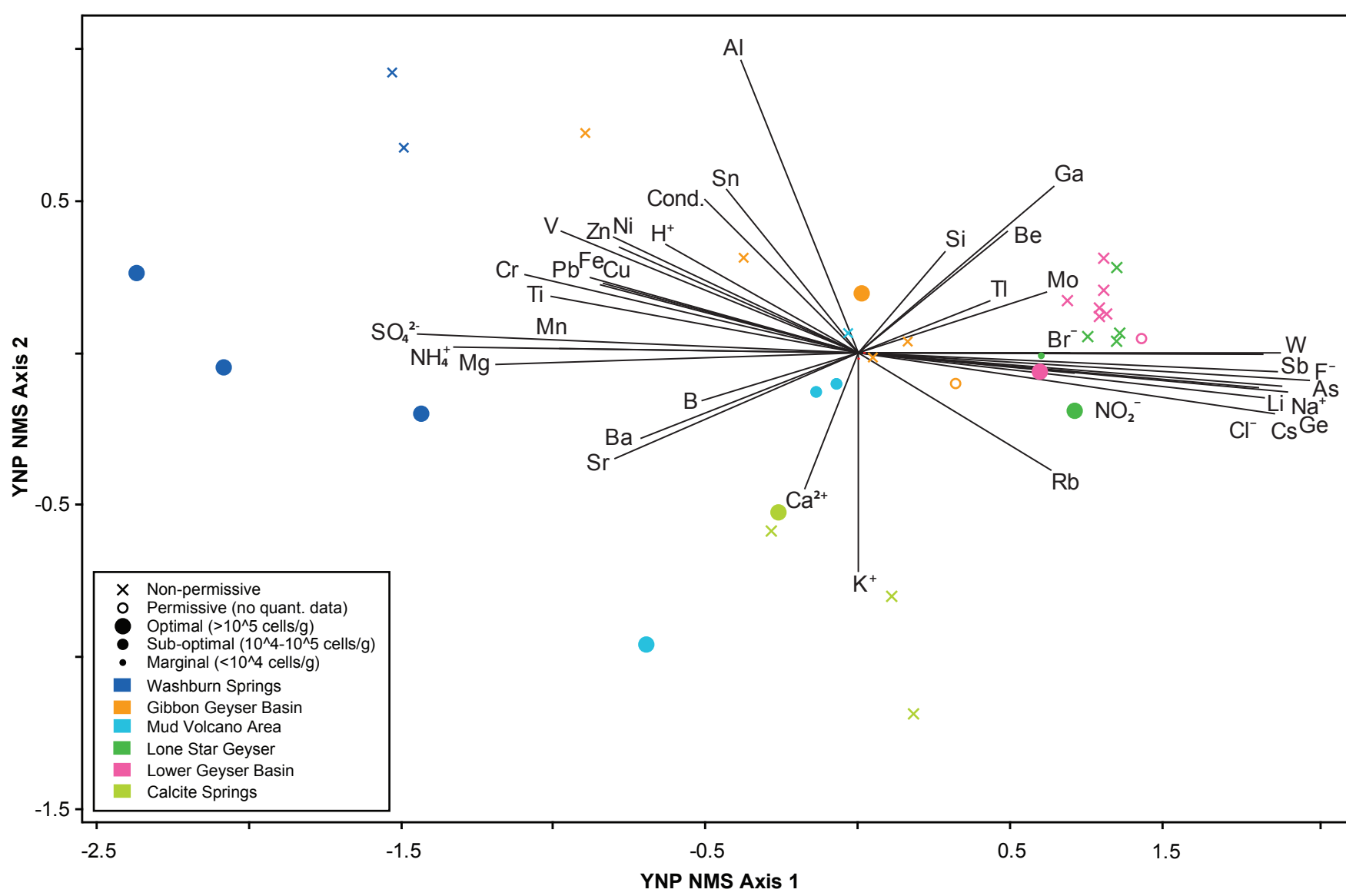

Supplement: Figure S6 — An NMS plot shows relationships among multiple geochemical variables from YNP sites. The ordination of geochemical analytes from the YNP samples yielded a reliable, two-axis solution (stress = 7.272; p = 0.0196; cumulative r2 = 0.975). Distance between sample sites is proportional to dissimilarity in geochemical composition. Geochemically similar sites cluster closely together, as shown by the separation of sites from different geothermal regions. Vectors in black illustrate correlations of individual analytes to ordination axes and are directed toward samples in which those analytes are elevated. The magnitude of these relationships is indicated by the length of the vectors, with the longest lines corresponding to the strongest relationships. Only r2≥0.2 are shown. H+ was determined from field pH measurements and reflects the activity of H+ (aH+) and not concentration. (PDF) [file pone.0035964.s006.pdf]

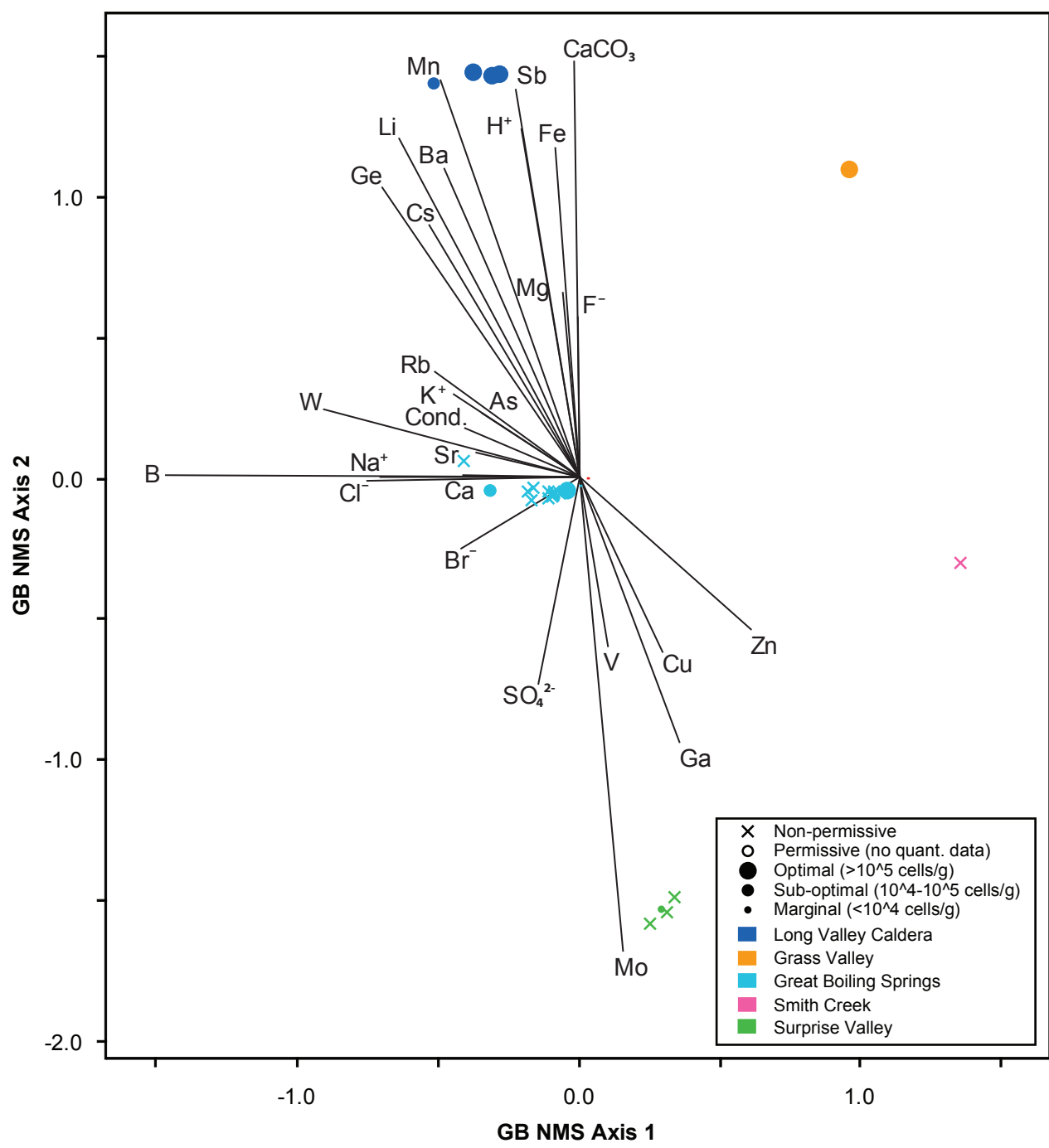

Supplement: Figure S7 — An NMS plot shows relationships among multiple geochemical variables from GB sites. The ordination of geochemical analytes from the GB samples yielded a reliable, two-axis solution (stress = 5.176; p = 0.0196; cumulative r2 = 0.903). Distance between sample sites is proportional to dissimilarity in geochemical composition. Geochemically similar sites cluster closely together, as shown by the separation of sites from different geothermal regions. Vectors in black illustrate correlations of individual analytes to ordination axes and are directed toward samples in which those analytes are elevated. The magnitude of these relationships is indicated by the length of the vectors, with the longest lines corresponding to the strongest relationships. Only r2≥0.2 are shown. H+ was determined from field pH measurements and reflects the activity of H+ (aH+) and not concentration. (PDF) [file pone.0035964.s007.pdf]
