# Supplementary material for: Korarchaeota Diversity, Biogeography, and Abundance in Yellowstone and Great Basin Hot Springs and Ecological Niche Modeling Based on Machine Learning
Source: PLoS One. 2012 May 4;7(5):e35964. doi: 10.1371/journal.pone.0035964 (PMC3344838; doi:10.1371/journal.pone.0035964)
Supplement: Figure S2 — Korarchaeota abundance, as determined by qPCR, decreased with decreasing temperature along the Little Hot Creek outflow system (GB). Isotherms were modeled using point temperature data from the sample sites shown here. The only channels delineated are those for which abundance data were available. (PDF) [file pone.0035964.s002.pdf]

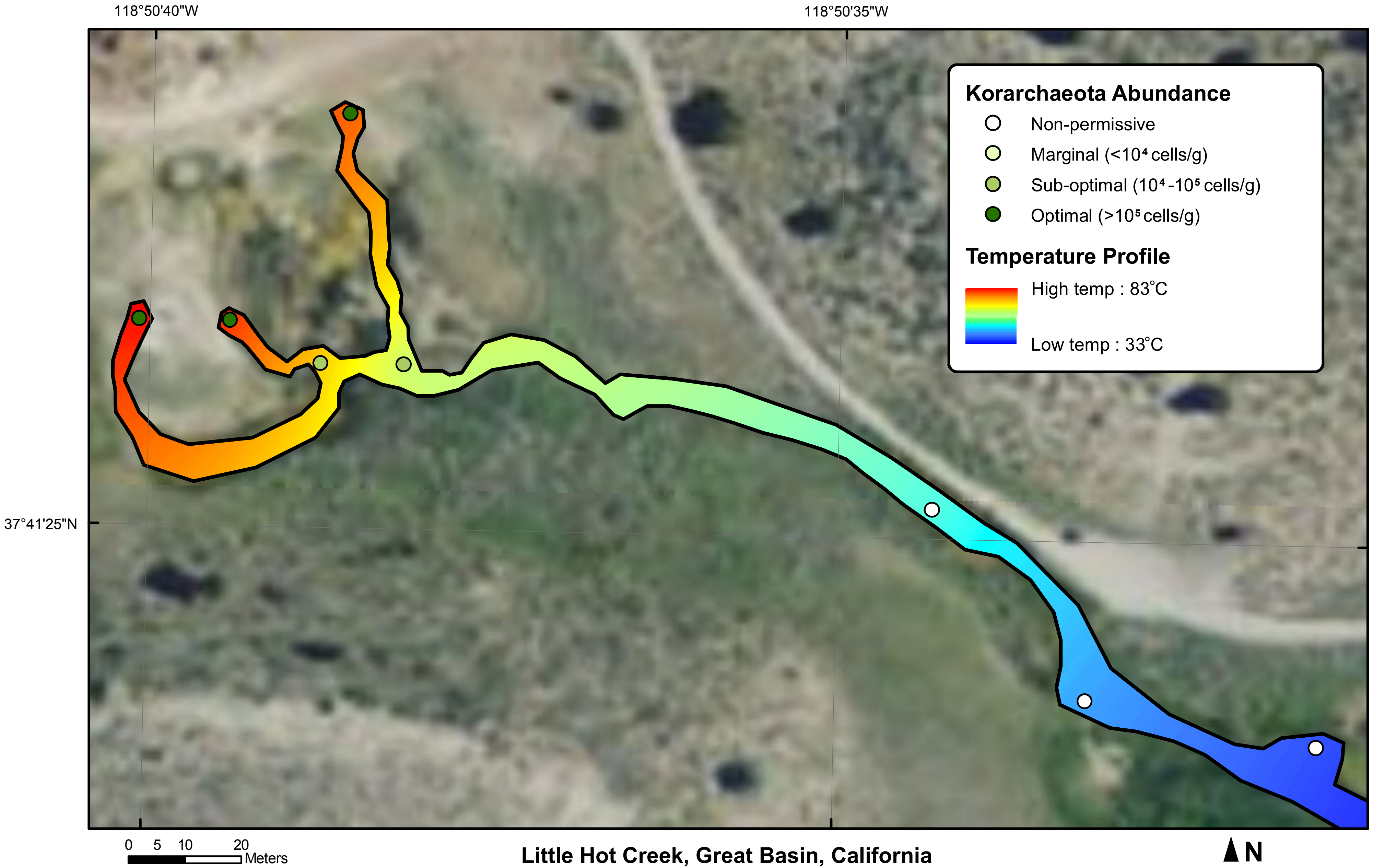

Little Hot Creek, Great Basin, California
