# Supplementary material for: Korarchaeota Diversity, Biogeography, and Abundance in Yellowstone and Great Basin Hot Springs and Ecological Niche Modeling Based on Machine Learning
Source: PLoS One. 2012 May 4;7(5):e35964. doi: 10.1371/journal.pone.0035964 (PMC3344838; doi:10.1371/journal.pone.0035964)
Supplement: Table S2 — Description of GB and YNP hot springs in which Korarchaeota 16S rRNA genes were not detected. (DOC) [file pone.0035964.s009.doc]

Table S2. Description of GB and YNP hot springs in which *Korarchaeota* 16S rRNA genes were not detected.

|  |  |  |  |  |  | Temp |  |
| --- | --- | --- | --- | --- | --- | --- | --- |
| Hot Spring Name | | Thermal Region | Thermal Area | GPS | sample type (mineralogy)^a^ | (˚C) | pH |
| Great Basin | |  |  |  |  |  |  |
|  | SC1 | Smith Creek | Smith Creek | 39º18.816'N 117º32.778'W | NA | 77.6 | 7.20 |
|  | GBS | Great Boiling Springs | Great Boiling Sprgs | 40º39.689'N 119º29.968'W | brown mud (S,I,K,Q,Z) | 73.4 | 7.44 |
|  | GBS04b | Great Boiling Springs | Great Boiling Sprgs | 40º39.749'N 119º21.986'W | compact gray sediment, green/brown mat (S,I,K,Q,Z) | 76.8 | 7.62 |
|  | GBSX2 | Great Boiling Springs | Great Boiling Sprgs | 40°39.762'N 119º21.991'W | gray sediment, brown/red mat | 55.5 | 6.76 |
|  | GBS 09 | Great Boiling Springs | Great Boiling Sprgs | 40º39.778'N 119º21.996'W | gray/brown sediment, brown/gray/green mat | 45.8 | 7.77 |
|  | GBS C1 | Great Boiling Springs | Great Boiling Sprgs | 40º39.755'N 119º22.258'W | sediment, fluffy gray/brown mat | 48.7 | 7.56 |
|  | GBS 16 | Great Boiling Springs | Great Boiling Sprgs | 40º39.695'N 119º21.971'W | fine gray sediment, lt. brown mat | 60.7 | 7.75 |
|  | GBS 15 | Great Boiling Springs | Great Boiling Sprgs | 40º39.709'N 119º21.965'W | brown mud | 67.7 | 7.59 |
|  | GBS X3 | Great Boiling Springs | Great Boiling Sprgs | 40º39.772'N 119º21.989'W | gray sediment, brown/orange mat | 58.4 | 7.18 |
|  | GBS 10 | Great Boiling Springs | Great Boiling Sprgs | 40º39.794'N 119º21.998'W | fluffy gray sediment, brown/green/orange mat | 52.5 | 7.23 |
|  | GBS 19 | Great Boiling Springs | Great Boiling Sprgs | 40º39.763'N 119º22.017'W | rocky brown/gray hard sediment | 90.7 | 7.22 |
|  | SSE | Great Boiling Springs | Sandy Springs | 40º39.173'N 119º22.471'W | gray muddy sediment (S,I,K,Q,KF) | 77.0 | 7.30 |
|  |  | | | | | | |
|  | SSWcon3 | Great Boiling Springs | Sandy Springs | 40º39.121'N 119º22.465'W | clumpy charcoal & lt. brown sediment, green gel-like mat | 50.7 | 8.43 |
|  | SVX1 | Surprise Valley | Surprise Valley | 41º32.049'N 120º04.386 W | airy brown/gray sediment | 72.3 | 8.25 |
|  | SV2 | Surprise Valley | Surprise Valley | 41º32.042'N 120º04.367'W | lt. brown rocky mud | 83.5 | 8.18 |
|  | SVX3 | Surprise Valley | Surprise Valley | 41º32.085'N 120º04.385'W | brown/green mud | 80.0 | 8.29 |
|  | SV2con8 | Surprise Valley | Surprise Valley | 41º32.020'N 120º04.251'W | gray/lt. brown sediment, brown/green filaments | 56.8 | 8.73 |
|  | SV2con17 | Surprise Valley | Surprise Valley | 41º32.054'N 120º04.129'W | fine thick gray sediment, brown/green filaments | 49.2 | 8.93 |
|  | SV2con18 | Surprise Valley | Surprise Valley | 41º32.026'N 120º04.125'W | fluffy green/brown biomass | 38.6 | 8.93 |
|  |  | | | | | | |
|  | LHCcon3 | Long Valley Caldera | Little Hot Creek | 37º41.420'N 118º50.510'W | black mat | 51.0 | 7.94 |
|  | LHCcon4 | Long Valley Caldera | Little Hot Creek | 37º41.401'N 118º50.549'W | green/brown biomass | 43.0 | 8.23 |
|  | LHCcon5 | Long Valley Caldera | Little Hot Creek | 37º41.397'N 118º50.521'W | green/brown biomass | 37.1 | 8.26 |
| Yellowstone National Park | |  |  |  |  |  |  |
|  | 070714G | Gibbon Geyser Basin | Sylvan Springs | NA | off-white sand | 89.2 | 1.44 |
|  | 070714B | Gibbon Geyser Basin | Sylvan Springs | 44°41.969'N 110°46.070'W | gritty grey sand | 58.0 | 1.30 |
|  | Evening Primrose (EP) | Gibbon Geyser Basin | Sylvan Springs | 44°41.961'N 110°46.033'W | gray mud | 84.1 | 5.56 |
|  | 070714C | Gibbon Geyser Basin | Sylvan Springs | 44°42.001'N 110°45.894'W | gritty brown sand | 52.9 | 1.80 |
|  | 060805O | Gibbon Geyser Basin | Sylvan Springs | 44°41.971'N 110°46.028'W | light brown mud | 42.1 | 4.97 |
|  | 060809D | Washburn Hot Springs | "Washburn Area" | NA | brown mud | 85.2 | 3.05 |
|  | 060809F | Washburn Hot Springs | "Washburn Area" | NA | red/orange sediment & rocks | 83.3 | 3.17 |
|  | -NA- 060809 | Washburn Hot Springs | "Washburn Area" | NA | black sediment | 74.8 | 3.16 |
|  | Mound OF (Mnd OF) | Lower Geyser Basin | Sentinel Meadows Grp | 44°33.840'N 110°51.757'W | lt. brown rocks | 83.1 | 8.26 |
|  | Mound Cone OF 1 | Lower Geyser Basin | Sentinel Meadows Grp | 44°33.891'N 110°51.605'W | NA | 88.0 | 8.80 |
|  | Mound Cone OF 1.5 | Lower Geyser Basin | Sentinel Meadows Grp | 44°33.891'N 110°51.606'W | lg. particles, multicolor rocks | 79.5 | 8.88 |
|  | Mound Cone OF 2 | Lower Geyser Basin | Sentinel Meadows Grp | 44°33.887'N 110°51.610'W | off-white, rocky | 74.2 | 9.01 |
|  | Mound Cone OF 3 | Lower Geyser Basin | Sentinel Meadows Grp | 44°33.888'N 110°51.614'W | orange filaments | 70.0 | 9.20 |
|  | Mound Cone OF 4 | Lower Geyser Basin | Sentinel Meadows Grp | 44°33.888'N 110°51.615'W | orange filaments | 64.3 | 9.10 |
|  | 070710I | Lower Geyser Basin | Sentinel Meadows Grp | 44°34.177'N 110°51.906'W | lg. white rocks, green biomass | 93.6 | 7.81 |
|  | 070710J | Lower Geyser Basin | Sentinel Meadows Grp | 44°34.179'N 110°51.903'W | dark orange/red biomass | 83.5 | 8.06 |
|  | 070710K | Lower Geyser Basin | Sentinel Meadows Grp | 44°34.180'N 110°51.899'W | gray/green/clear filaments | 73.9 | 8.25 |
|  | 070710L | Lower Geyser Basin | Sentinel Meadows Grp | 44°34.180'N 110°51.897'W | green/orange biomass, gritty | 70.9 | 8.28 |
|  | 070710M | Lower Geyser Basin | Sentinel Meadows Grp | 44°34.184'N 110°51.894'W | green/orange biomass, gritty | 58.3 | 8.48 |
|  | Steep Cone (SC) | Lower Geyser Basin | Sentinel Meadows Grp | 44°33.888'N 110°51.744'W | lt. brown rocks, orange/brown filaments | 93.6 | 7.14 |
|  | Steep Cone (OF 1) | Lower Geyser Basin | Sentinel Meadows Grp | NA | orange filaments | 82.1 | 8.00 |
|  | Steep Cone (OF 2) | Lower Geyser Basin | Sentinel Meadows Grp | NA | orange filaments | 71.0 | 8.34 |
|  | Flatcone OF 1 (FC OF 1) | Lower Geyser Basin | Sentinel Meadows Grp | 44°34.110'N 110°51.812'W | orange/gray filaments | 78.4 | 8.45 |
|  | Flatcone OF 2 | Lower Geyser Basin | Sentinel Meadows Grp | NA | orange filament, green biomass | 67.6 | 8.58 |
|  | 070707P | Lower Geyser Basin | River Group | 44°33.528'N 110°50.635'W | black sand | 91.0 | 8.09 |
|  | 070707Q | Lower Geyser Basin | River Group | 44°33.526'N 110°50.628'W | black sand | 81.0 | 8.42 |
|  | 070707S | Lower Geyser Basin | River Group | 44°33.531'N 110°50.626'W | green/orange biomass, gritty | 65.5 | 8.63 |
|  | 070716O | Lower Geyser Basin | NA | 44°32.811'N 110°51.008'W | brown grit | 94.0 | 7.85 |
|  | 070716P | Lower Geyser Basin | NA | 44°32.807'N 110°51.002'W | brown grit | 81.6 | 8.22 |
|  | 070716Q | Lower Geyser Basin | NA | 44°32.795'N 110°51.008'W | lg. rocky sediment | 68.6 | 8.37 |
|  | 070716R | Lower Geyser Basin | NA | 44°32.792'N 110°51.005'W | orange/green biomass, sandy | 64.0 | 8.46 |
|  | 070716S | Lower Geyser Basin | NA | 44°32.786'N 110°50.999'W | orange/green biomass, lg. particles | 54.4 | 8.70 |
|  | Octopus Source (Oct Src) | Lower Geyser Basin | White Creek Group | 44°32.044'N 110°47.873'W | reddish gritty sand | 90.7 | 7.58 |
|  | Octopus OF 1 | Lower Geyser Basin | White Creek Group | 44°32.042'N 110°47.882'W | orange clumpy biomass | 80.9 | 7.79 |
|  | Octopus OF 2 | Lower Geyser Basin | White Creek Group | 44°32.046'N 110°47.885'W | off-white, rocky | 70.0 | 8.08 |
|  | Octopus OF 3 | Lower Geyser Basin | White Creek Group | 44°32.046'N 110°47.899'W | lg. white rocks, orange/red/black biomass | 65.0 | 8.25 |
|  | Octopus OF 4 | Lower Geyser Basin | White Creek Group | 44°32.045'N 110°47.896'W | green/orange biomass | 54.8 | 8.41 |
|  | 070706F | Lone Star | NA | NA | brown sand | 87.9 | 8.11 |
|  | 070706G | Lone Star | NA | NA | orange filaments | 77.1 | 8.27 |
|  | 060808G_1 | Lone Star | NA | NA | brown/gray mud | 88.5 | 7.61 |
|  | 060808G | Lone Star | NA | NA | lt. brown rocks | 75.7 | 7.72 |
|  | 070707Y | Lone Star | NA | NA | lg. beige/multicolor rocks | 77.8 | 7.95 |
|  | 070707Z | Lone Star | NA | NA | lg. beige/multicolor rocks | 67.6 | 8.21 |
|  | 070706H | Lone Star | NA | NA | beige sand | 81.0 | 7.08 |
|  | 070715B | Mud Volcano Area | "GOPA" | NA | gray grit, fine brown sediment | 65.7 | 4.55 |
|  | 060811Y | Calcite | Calcite | 44°27.751'N 110°51.294'W | black sediment | 72.3 | 6.57 |
|  | 070712X | Calcite | Calcite | NA | steel gray sand | 94.3 | 8.28 |
|  | 070712V | Calcite | Calcite | NA | gritty gray sand | 75.6 | 8.11 |
|  | By The Rock | Calcite | Calcite | NA | black sediment | 73.2 | 6.67 |

^a^ Minerals detected, S, smectite; I, illite; K, kaolinite; Q, quartz; KF, potassium feldspar; PF, plagioclase feldspar; CA, carbonate apatite; Z, zeolite clinoptilolite; C, calcite.

NA, not available
