# Supplementary material for: Korarchaeota Diversity, Biogeography, and Abundance in Yellowstone and Great Basin Hot Springs and Ecological Niche Modeling Based on Machine Learning
Source: PLoS One. 2012 May 4;7(5):e35964. doi: 10.1371/journal.pone.0035964 (PMC3344838; doi:10.1371/journal.pone.0035964)
Supplement: Table S3 — Particulate geochemistry of selected springs and summary of statistics relating analytes to Korarchaeota presence and abundance in selected Yellowstone springs. (DOCX) [file pone.0035964.s010.docx]

TABLE S3. Particulate geochemistry of selected springs and summary of statistics relating analytes to *Korarchaeota* presence and abundance in selected Yellowstone springs^a^.

|  | **Carbon** | | | | | **Nitrogen** | |
| --- | --- | --- | --- | --- | --- | --- | --- |
|  | **C_Total_**  **(wt. %)** | **C_Org_**  **(wt. %)** | **C_Inorg_**  **(wt. %)** | **δ^13^C_Total_**  **(‰)** | **δ^13^C_Org_**  **(‰)** | **N_Total_**  **(wt. %)** | **N_Org_**  **(wt. %)** |
| Permissive (abundance)^b^ | |  |  |  |  |  |  |
| 070714A (O) | 0.99 ± 0.002 | 0.98 | 0.01 | -20.88 ± 0.01 | -20.90 | 0.12 ± 0.0004 | 0.11 |
| 060809E (O) | 0.23± 0.001 | 0.23 | 0.00 | -13.47 ± 0.26 | -13.65 | 0.36 ± 0.001 | 0.37 |
| 060809A (O) | 0.34 | 0.33 ± 0.004 | 0.00 | -22.02 | -22.52 ± 0.09 | 0.34 | 0.34 ± 0.0004 |
| Non-permissive | |  |  |  |  |  |  |
| 060808G _1 | 0.19 ± 0.004 | 0.18 | 0.01 | -19.21 ± 0.28 | -19.74 | 0.02 ± 0.001 | 0.02 |

^a^ _­_Carbon and nitrogen content are expressed as weight percent (wt. %), C and N isotopic compositions are expressed in permil (‰) relative to PDB and air standards, respectively. C_Inorg_ (wt. %) was calculated by difference (C_Inorg_ = C_total_  - C­_org_). Most particulate geochemistry measurements were made in duplicate; error values are ±1 standard deviation (S.D.); the errors reflect sample heterogeneity and, thus, are sometimes larger than the analytical uncertainty for these measurements (generally, <0.2% for mass and ~0.02‰ for isotopic compositions). ^b^ Abundance defined as O is “optimal”, >10^5^ cells/g
